# Coregulation of transcription factors and microRNAs in human transcriptional regulatory network

**DOI:** 10.1186/1471-2105-12-S1-S41

**Published:** 2011-02-15

**Authors:** Cho-Yi Chen, Shui-Tein Chen, Chiou-Shann Fuh, Hsueh-Fen Juan, Hsuan-Cheng Huang

**Affiliations:** 1 Institute of Biomedical Informatics, Center for Systems and Synthetic Biology, National Yang-Ming University, Taipei, Taiwan; 2 Institute of Biological Chemistry, Academia Sinica, Taipei, Taiwan; 3 Genome and Systems Biology Degree Program, Department of Life Science, Institute of Molecular and Cellular Biology, Graduate Institute of Biomedical Electronics and Bioinformatics, National Taiwan University, Taipei, Taiwan; 4 Department of Computer Science and Information Engineering, National Taiwan University, Taipei, Taiwan

## Abstract

**Background:**

MicroRNAs (miRNAs) are small RNA molecules that regulate gene expression at the post-transcriptional level. Recent studies have suggested that miRNAs and transcription factors are primary metazoan gene regulators; however, the crosstalk between them still remains unclear.

**Methods:**

We proposed a novel model utilizing functional annotation information to identify significant coregulation between transcriptional and post-transcriptional layers. Based on this model, function-enriched coregulation relationships were discovered and combined into different kinds of functional coregulation networks.

**Results:**

We found that miRNAs may engage in a wider diversity of biological processes by coordinating with transcription factors, and this kind of cross-layer coregulation may have higher specificity than intra-layer coregulation. In addition, the coregulation networks reveal several types of network motifs, including feed-forward loops and massive upstream crosstalk. Finally, the expression patterns of these coregulation pairs in normal and tumour tissues were analyzed. Different coregulation types show unique expression correlation trends. More importantly, the disruption of coregulation may be associated with cancers.

**Conclusion:**

Our findings elucidate the combinatorial and cooperative properties of transcription factors and miRNAs regulation, and we proposes that the coordinated regulation may play an important role in many biological processes.

## Background

Transcriptional regulatory networks describe the interactions between transcriptional regulatory proteins and their target genes [1-3]. These regulators, known as transcription factors (TFs), are proteins that bind to specific DNA sequences and thereby control the transcription of genetic information encoded in DNA sequences. The interactions between TFs and target genes regulate the transcriptional activities of genome and thus determine the global gene expression program of a living cell.

In the last decade, microRNAs (miRNAs) have emerged as another prominent class of gene regulators. miRNAs are endogenous small RNA molecules that are abundant in animals, plants, and some viruses. They can reduce stability and/or translation activity of fully or partially sequence-complementary messenger RNAs (mRNAs), thus regulating gene expression at the post-transcriptional level. It has been found that miRNAs may control many biological processes in development, differentiation, growth, and even cancer development and progression [4-6].

Recent studies have suggested that miRNAs and TFs are primary metazoan gene regulators, and they seem to function in a similar regulatory logic, such as pleiotropy, combinatorial and cooperative activity, regulation, and even network motifs [7,8]. However, how miRNAs interplay and coordinate with TFs in the regulatory network still remains unclear. Since combinatorial interactions between miRNAs and TFs are complicated and thus hard to be validated by high-throughput experiments, computational modelling may provide a better clue to understand such complex relationships.

Currently, to uncover the coregulation interactions between miRNAs and TFs, researchers have to overcome two challenges. One is the incomplete knowledge of regulatory targets. Because the available experimentally verified targets of miRNAs and TFs are far from complete, the regulatory target datasets for global analysis were mainly from computational prediction. The other challenge is about how to integrate transcriptional and post-transcriptional layers to discover highly confident coregulation relationships. To solve these problems, previous studies have developed a bottom-up strategy; that is, they inferred the coordination between two upstream regulators from their downstream shared targets [9,10]. These inferences were basically based on different probabilistic models and statistical tests to measure the significance of shared targets between regulators. Indeed, the methods successfully eliminated those insignificant coregulation interactions occurred merely by chance; however, the biological meanings were ignored in the integration of transcriptional and post-transcriptional regulation interactions.

Here we proposed a novel framework utilizing functional annotation information to identify significant coregulation between transcriptional and post-transcriptional layers. Based on this model, function-enriched coregulation pairs were discovered, and the regulators were subsequently linked by enriched functions. With these functional linkages, we further constructed functional coregulation networks between regulators and investigated their characteristics. Next, we searched for the network motifs consisting of those function-enriched coregulation pairs, and found that an abundance of pairs were closely connected in their upstream. Finally, the expression patterns of function-enriched coregulation pairs were analyzed. Different coregulation types showed distinct expression correlation trends. More importantly, we found that the disruption of coregulation may be closely related to cancers.

## Methods

### Regulation relationships

The transcriptional regulation relationships between human transcription factors and their target genes were collected from TRED (Transcriptional Regulatory Element Database) [11]. The database provides genome-wide promoter annotation and transcription factor binding information from computational prediction and experimental evidence.

To collect all human TF-target regulation relationships in TRED, we firstly queried the list of all human TFs in the database. A total of 178 human TFs were obtained by this step. Next, we searched TF target genes for each TF using default parameters (promoter quality from "known, curated" to "with RNA" and "all" binding quality). The results showed that only 133 TFs were found to have at least one target gene by these criteria, and the final number of unique TF-target relationships was 6,764, which were used to construct the human transcriptional regulatory network for our analysis.

Since the available experimentally verified human miRNA targets are far from complete and thus not enough for global analysis, we used predicted miRNA targets from the TargetScan database (release 4.2) to perform the analysis [12]. In addition, different mature miRNAs may have identical seed regions and thereby target the same binding sites. To eliminate those coregulation interactions among the miRNAs with identical seed regions, we grouped mature miRNAs into families based on the miRNA family information from TargetScan. A total of 162 miRNA families and 7,521 target genes with 44,782 interactions were collected.

It is still difficult to predict the promoter region of miRNA genes in the genome. But it has been known that embedded miRNAs frequently coexpress with their host genes [13,14]. Therefore, we extracted miRNA host gene information from miRBase [15] and integrated the embedded miRNAs biogenesis information into the established transcriptional regulation network. A total of 310 premature miRNAs were found embedded in 259 host genes. Most of them (93%) were resided in introns.

### Identification of significant coregulation relationships

**Figure 1:**
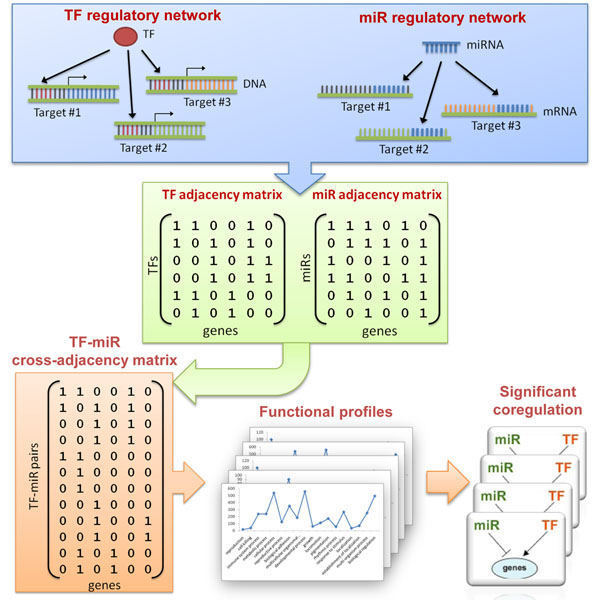
Example workflow for identifying significant coregulation

Combing all the potential targets of miRNAs and TFs, we firstly constructed two adjacency matrixes describing the regulator-target interaction for TFs and miRNAs, respectively. Then the two matrixes were combined into three cross-adjacency matrixes representing the shared targets of TF-TF, miRNA-miRNA, and TF-miRNA coregulation pairs. An example of identification of TF-miRNA coregulation pairs is shown in Figure 1.

Secondly, for each group of shared targets, the distribution of Gene Ontology (GO) annotations [16] at the second level in the biological process namespace was calculated. We chose the second level ontology because most of the genes were generally well-annotated at this level and these annotations provided a good balance between the sensitivity and the specificity in the following functional enrichment test. The distributions were considered as the functional profiles or fingerprints for these coregulation pairs.

Next, we utilized a randomization method to perform a permutation test for functional enrichment. For each group of shared targets, we randomly selected a null group of the same size from whole human genome as background. After 10,000 iterations, the log-likelihood score under multivariate hypergeometric distribution was measured to quantify the significance of functional enrichment. The correction for multiple comparisons was made under 0.05 false discovery rate (FDR) [17]. The final results of significant coregulation pairs were listed in additional file 1.

### Functional linkages and networks

**Figure 2:**
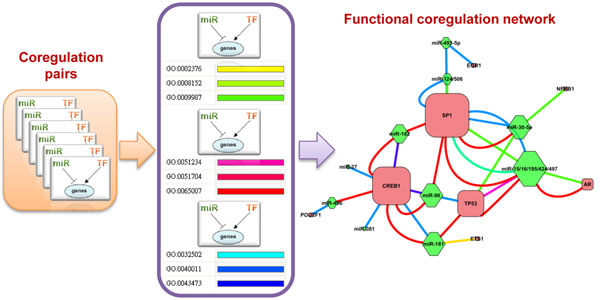
**Example workflow for constructing functional coregulation network**. For each coregulation pair, a function linkage was established if a GO term enriched in their shared targets. The coregulation network was generated based on these function linkages. Nodes represent regulators, and edge represents GO terms, marked with different colours.

For each function-enriched coregulation pair, Fisher’s Exact Test following FDR correction were conducted to identify enriched GO terms. Similarly, we only focused on the second level terms in biological process namespace. A functional linkage was established if a GO term overrepresented in the shared targets of a coregulation pair, implying that the two paired regulators may function coordinately in the specific biological process. Based on these linkages, we further constructed the functional coregulation networks (Figure 2). In order to investigate the specificity of coregulation relationships and provide a global view, only those linkages with relatively high significance that passed FDR-BL correction [17] were used to construct the networks.

### Enriched network motifs

We searched for network motifs preferentially occurred in function-enriched coregulation pairs rather than in random pairs by a resampling process. The predicted TF-targeting interactions for miRNA genes were collected from miRBase [15] and from literature [9]. In addition, we assumed that those embedded miRNA genes have same transcription units as their host genes and would be regulated together.

A total of 10,000 background sets of regulator pairs that have the same size as the set of function-enriched pairs were randomly selected from the global network. For each type of network patterns (sub-graphs), the observed frequency from the function-enriched coregulation pairs was first calculated and compared to the background distribution for assessment of significance. Only those network patterns with occurrence probabilities less than 0.001 were considered significant motifs (see additional file 2 for these significant motifs).

### Analysis of expression profiles

The miRNA and mRNA expression profiles were adopted from a previous study [18]. A total of 217 miRNAs and ~16,000 mRNAs across 8 human tissues (colon, pancreas, kidney, bladder, prostate, uterus, lung, and breast) were measured using miRNA bead-arrays and mRNA microarrays. Both normal and tumor samples were profiled for each tissue. For each type of coregulation, we first generated background distribution by calculating the Pearson's correlation coefficients (PCCs) of expression profiles between the two paired regulators in all possible pairs (i.e., those pairs shared no targets and/or those pairs not identified as function-enriched). After that, the distribution of enriched coregulation pairs was calculated and shown against the background.

## Results

### Functional coregulation pairs

After the integration of miRNA regulation into human transcriptional regulation network, we adopted a novel strategy utilizing functional information to identify function-enriched coregulation pairs, and establish function linkages for each pair. Traditional analysis of functional enrichment was aimed at elucidating the regulatory roles of each individual regulator only, inevitably leaving some significant coregulation hidden in the traditional views. Instead, based on our model, different regulation types involving single regulators or combinations of regulators can all be studied and compared.

**Figure 3:**
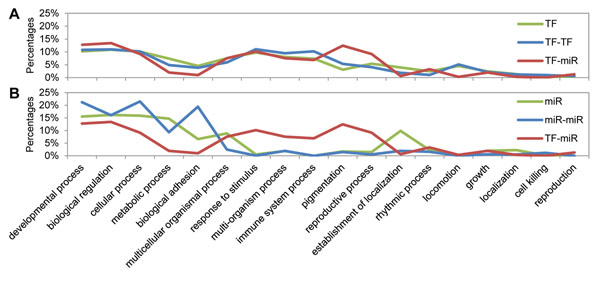
**Distributions of enriched biological processes for different regulation types**. (A) Distributions for TF-involving regulation: individual TFs, TF-TF pairs, and TF-miRNA pairs. (B) Distributions for miRNA-involving regulation: individual miRNAs, miRNA-miRNA pairs, and TF-miRNA pairs. Note that the same TF-miRNA line are drawn in both (A) and (B) for comparison.

The distributions of different regulation types were grouped into two diagrams for comparing. Figure 3A shows distributions of individual TF regulation and TF-TF coregulation. The two distributions look similar; however, two biological processes, pigmentation and reproductive process, emerge when it comes to TF-miRNA coregulation, implying that the two biological processes may be the typical processes in which TFs should coordinate with miRNAs to control the expression programs.

In contrast, miRNA-involving regulation shows divergent distributions in Figure 3B. The top ranked biological processes of individual miRNA regulation were biological regulation, cellular process, and developmental process, which were the previously known miRNA-involving processes [4-6]. On the other hand, biological adhesion was relatively high in miRNA-miRNA coregulation, suggesting that miRNAs may regulate this process majorly in a coordination manner.

Moreover, many biological processes enriched in TF-miRNA coregulation were relatively poor in the regulation involving miRNAs only. In other words, those processes may be the typical processes needed to be coordinately regulated by TFs and miRNAs, and the coordination may provide a mechanism to switch expression programs. More importantly, it suggested that, by coordinating with TFs, miRNAs may engage in a wider diversity of biological processes, and these undiscovered processes were failed to be identified by traditional analysis of functional enrichment for a single regulator.

### Functional coregulation networks

In the previous section, different regulators were connected by identified functional linkages, which represented that the two paired regulators may function in coordination with each other in a specific biological process. We further built up functional coregulation networks from these linkages and found interesting properties in the networks.

**Figure 4:**
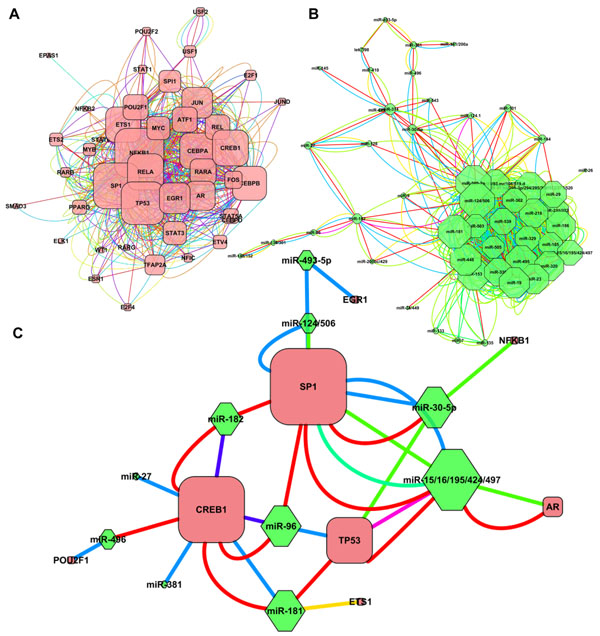
**Functional coregulation networks.** (A) TF-TF coregulation network. (B) miR-miR coregulation network. (C) TF-miR coregulation network. Red nodes represent TFs; green nodes represent miRNAs; and edges represent biological processes. Different edge colours represent different biological processes.

Figure 4 shows the three subnetwork examples (the largest connected component) for different coregulation types. Obviously, it appears that the TF-TF coregulation network (Figure 4A) was in high density coregulation (avg. 3.52 links per pair) and full of diversity in edge types (avg. 5.70 edge types per regulator); in other words, TFs may function together in many kinds of biological processes. Likewise, the miRNA-miRNA coregulation network (Figure 4B) showed similar high density coregulation (avg. 3.12 links per pair) but the diversity of involving processes was lower (avg. 3.92 edge types per regulator) than TF-TF coregulation. In contrast, the TF-miRNA coregulation network (Figure 4C) was in higher specificity (avg. 1.48 links per pair; avg. 2.22 edge types per regulator); that is, a TF may coordinate with different neighbour miRNAs in specific biological processes, and *vice versa*. These results suggested that the cross-layer coregulation may have higher specificity than intra-layer coregulation.

### Network motifs for coregulation pairs

Many studies have been devoted to understanding network structures in gene regulatory networks, and have found that most networks seem to be largely composed of occurring patterns, called network motifs. The functions associated with common network motifs, such as auto-regulation and feed-forward loops (FFLs), were discovered and revealed by several researches both theoretically and experimentally [1,9,10,19-22].

**Figure 5:**
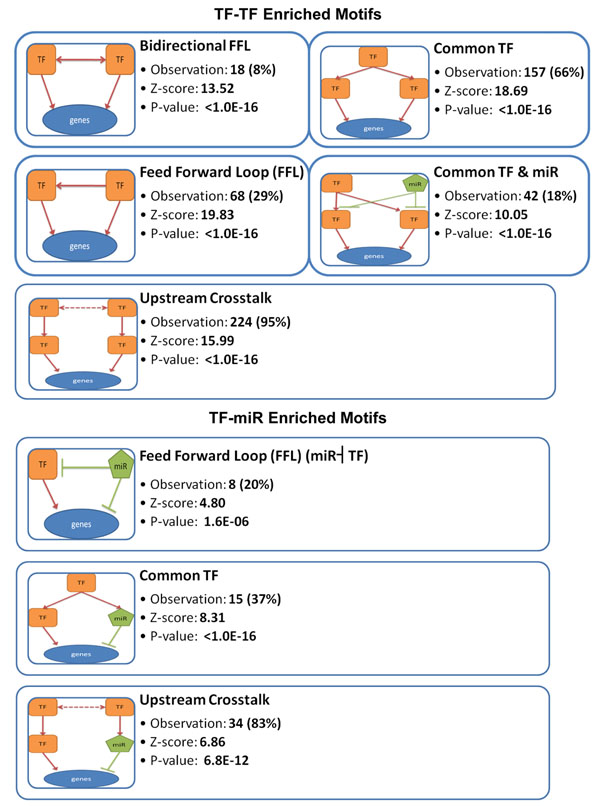
**Network motifs for TF-TF and TF-miRNA coregulation pairs**. For each motif, the observation number and the percentage of pairs that have this motif were reported, along with the *P*-value and one instance coregulation pair.

Unsurprisingly, the function-enriched coregulation pairs also have preferentially recurring network motifs as shown in Figure 5. Several types of motifs were found in TF-TF coregulation; for example, bidirectional and unidirectional FFLs were explored and these results were consistent with previous studies on network biology. In addition to FFLs, we went further to investigate the upstream regulatory patterns of coregulation pairs and found that the two paired regulators were closely linked in their upstream. For instance, over half of the pairs had common upstream TFs; a significant number of pairs had common TFs and miRNAs; and almost all pairs were cross-regulated in their upstream. Similar results were also found in TF-miRNA coregulation. However, no enriched motif was found in miRNA-miRNA coregulation, probably due to the incomplete knowledge of regulatory regions of miRNA genes.

### Expression patterns of coregulation pairs

Expression data across human normal/tumor tissues have recently become available. A previous study measured miRNA and mRNA expression profiles across 8 tissues (colon, pancreas, kidney, bladder, prostate, uterus, lung, and breast) and each tissue contained both normal and tumor samples [18]. By analyzing the expression profiles, we investigated the correlations between the expression profiles of each coregulation pair in both normal and cancer samples.

**Figure 6:**
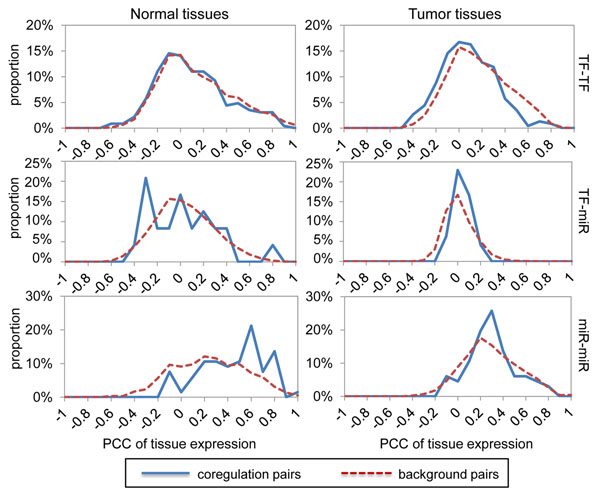
**Distributions of expression correlation for different types of coregulation pairs.** Left column for normal tissues; right column for tumor tissues. Rows are in the order of TF-TF, TF-miR, and miR-miR coregulation pairs.

Figure 6 shows the expression correlations of different coregulation types in normal and tumor samples. Interestingly, the three coregulation types show distinct trends in normal tissues. For example, TF-TF had a zero-centered distribution similar to background; TF-miRNA had two tendencies to highly-positive and medium-negative correlations in comparison with its background; miRNA-miRNA showed preference for only positive correlation. The different trends may reflect the diversity of function roles between TFs and miRNAs; that is, TFs can act as activators (+) or repressors (-) in gene regulation; however, miRNAs mainly act as repressors (-) by translation inhibition or transcript destabilization. Thus, it seems that these typical trends may be mechanistically reasonable.

On the contrary, all coregulation types turn into an identical trend in tumor tissues. All of them show similar zero-centered distributions resembled to their backgrounds. This trend suggests that the function-enriched coregulation pairs lost their correlation in tumor tissues, implying the disruption of coregulation may be closely associated to cancers. Together these results may support the functionality of identified coregulation pairs.

## Discussion and conclusion

We proposed a novel strategy aimed at identifying potential coordinated regulation by utilizing functional annotation information and discovered many biological processes that emerged only in coregulation. Compared to traditional function enrichment analysis, our strategy considered whole function profiles rather than single annotations. In addition, it also solves the restriction of traditional methods that only focus on single regulator. For example, a miRNA can potentially regulate an abundance of target genes. To find enriched functions of the miRNA, all its potential targets will be tested for any enriched function. However, since the target size of a miRNA may be huge, some meaningful biological processes involving only a small subset of genes will be hidden. In fact, these hidden processes may be significantly impacted by miRNAs in coordination with other regulators, namely, other miRNAs or TFs. After all, a biological process may be regulated not only at the transcriptional layer, but also at the posttranscriptional layer [7,8,23,24].

Interestingly, our results show that pigmentation and reproductive process are two typical biological processes specifically emerging in TF-miRNA coregulation. It is suggested that miRNAs may provide genetic switch mechanisms to essentially inactivate the target genes, thus leading to detectable phenotypic consequences. In model organisms, there have been many studies investigating the switch-like role of miRNAs in pigmentation. For example, miRNAs can regulate the eye pigmentation genes in Drosophila [25]. The influence of miRNAs on pigmentation in zebrafish was also reported [26]. Another study found that miR-434-5p may mediate skin whitening and lightening in mouse [27]. And in melanoma cell lines, it is shown that miR-137 may target a pigmentation regulator [28].

The analysis of functional coregulation networks provided other clues. We found that a TF may regulate in coordination with different miRNAs in different biological processes, and *vice versa*. It suggested that the cross-layer coregulation may have higher specificity than intra-layer coregulation.

We also performed network motif analysis to see if any recurring pattern exists in coregulation network structure. Different types of feed-forward loops were found in TF-TF and TF-miRNA coregulation, and these results were consistent with several previous studies on transcriptional network [1,9,10,19-22]. Among these FFLs, a special kind of miRNA-mediated FFLs emerged in TF-miRNA coregulation. In this kind of FFLs, a miRNA may simultaneously repress a TF and its target genes, thus contributing to a switch-like control of expression programs. More importantly, we go further this time to investigate the upstream structure of coregulation pairs and found closely interaction in their upstream. It implies that the network structures of coregulation may have extensive crosstalk in the higher levels.

Finally, the expression analysis of coregulation discovered distinct trends in different coregulation types; namely, TF-TF showed no correlation, whereas miRNA-miRNA had a preference of positive correlation, and TF-miRNA appeared both positive and negative correlation. A previous study investigated only TF-miRNA correlation and found the same tendencies [9]. The authors rationalized this trend by pointing out the distinct function roles that TFs and miRNAs may play. We further supported this idea by showing the results of TF-TF and miRNA-miRNA coregulation, which were also consistent with the same interpretation. In addition, TF activities are under control at protein level; that is, TFs may be activated or deactivated by a number of mechanisms including phosphorylation, ligand binding, and interaction with other regulatory proteins. Therefore, it is not surprising that co-function TFs may show no correlation in mRNA expression level. Notably, a large proportion of TF-miRNA pairs showed negative correlation in expression profiles, which could be explained by the structure of the miRNA-mediated-FFLs discussed before, supporting the idea that many miRNAs in TF-miRNA coregulation contributed to switch-like regulation.

More significantly, by comparing the expression correlations between normal and tumor tissues, we found a common trend in function-enriched coregulation pairs; that is, the function-enriched pairs lost their correlation in tumor tissues. It suggested that the disruption of coregulation may lead to abnormal expression programs and may be directly associated to cancers.

Our findings shed light on the coregulation of miRNAs in transcriptional regulatory network. Future experimental works will provide more complete knowledge in transcriptional network and miRNA regulation, thus allowing the elucidation of more precise co-regulatory mechanisms.

## Competing interests

The authors declare that they have no competing interests.

## Authors' contributions

CYC implemented the computational method, carried out the analysis, and drafted the manuscript. CYC and HCH conceived of the study. STC and CSF helped to perform the analysis and participated in discussions of the research. HFJ and HCH participated in the design and coordination of the study, and edited the manuscript. All authors read and approved the final manuscript.

## Supplementary Material

Additional file 1 **List of all function-enriched coregulation pairs**http://idv.sinica.edu.tw/joeychen/APBC2011/AdditionalFile1.pdf Click here for file

Additional file 2 **List of network motifs for function-enriched coregulation pairs**http://idv.sinica.edu.tw/joeychen/APBC2011/AdditionalFile2.pdf Click here for file
