# Supplementary material for: Coregulation of transcription factors and microRNAs in human transcriptional regulatory network
Source: BMC Bioinformatics. 2011 Feb 15;12(Suppl 1):S41. doi: 10.1186/1471-2105-12-S1-S41 (PMC3044298; doi:10.1186/1471-2105-12-S1-S41)
Supplement: Additional file 1 — List of all function-enriched coregulation pairshttp://idv.sinica.edu.tw/joeychen/APBC2011/AdditionalFile1.pdf [file 1471-2105-12-S1-S41-S1.pdf]

Function-enriched Coregulation Pairs

A. TF-TF Coregulation

TF A	TF B	Enriched GO IDs
AR	ATF1	GO:0002376; GO:0009987; GO:0032501; GO:0032502; GO:0043473; GO:0050896; GO:0051704; GO:0065007
AR	CEBPA	GO:0001906; GO:0002376; GO:0008152; GO:0009987; GO:0032502; GO:0040011; GO:0050896; GO:0051234; GO:0051704; GO:0065007
AR	CEBPB	GO:0001906; GO:0002376; GO:0008152; GO:0009987; GO:0032502; GO:0050896; GO:0051234; GO:0051704; GO:0065007
AR	CREB1	GO:0002376; GO:0008152; GO:0009987; GO:0022414; GO:0032501; GO:0032502; GO:0050896; GO:0051704; GO:0065007
AR	EGR1	GO:0002376; GO:0009987; GO:0022414; GO:0032501; GO:0032502; GO:0040011; GO:0050896; GO:0051234; GO:0051704; GO:0065007
AR	ETS1	GO:0002376; GO:0009987; GO:0032501; GO:0032502; GO:0043473; GO:0050896; GO:0065007
AR	NFKB1	GO:0002376; GO:0008152; GO:0009987; GO:0032501; GO:0032502; GO:0040007; GO:0040011; GO:0043473; GO:0050896; GO:0051234; GO:0051704; GO:0065007
AR	POU2F1	GO:0001906; GO:0002376; GO:0008152; GO:0009987; GO:0032502; GO:0040011; GO:0043473; GO:0050896; GO:0065007
AR	RARA	GO:0002376; GO:0008152; GO:0009987; GO:0022414; GO:0032501; GO:0032502; GO:0040011; GO:0050896; GO:0051234; GO:0051704; GO:0065007
AR	REL	GO:0002376; GO:0009987; GO:0022610; GO:0032502; GO:0050896; GO:0051704; GO:0065007
AR	RELA	GO:0002376; GO:0008152; GO:0009987; GO:0032502; GO:0040007; GO:0043473; GO:0050896; GO:0051704; GO:0065007
AR	SP1	GO:0001906; GO:0002376; GO:0008152; GO:0009987; GO:0022414; GO:0032501; GO:0032502; GO:0040007; GO:0043473; GO:0050896; GO:0051704; GO:0065007
AR	SPI1	GO:0002376; GO:0009987; GO:0022610; GO:0040011; GO:0050896; GO:0051704; GO:0065007
AR	STAT3	GO:0002376; GO:0009987; GO:0032502; GO:0043473; GO:0050896; GO:0051704; GO:0065007
AR	STAT5A	GO:0002376; GO:0009987; GO:0032502; GO:0043473; GO:0050896; GO:0051704; GO:0065007
AR	TP53	GO:0002376; GO:0008152; GO:0009987; GO:0022414; GO:0032501; GO:0032502; GO:0040007; GO:0040011; GO:0043473; GO:0050896; GO:0051704; GO:0065007
ATF1	CEBPA	GO:0002376; GO:0008152; GO:0009987; GO:0022610; GO:0032501; GO:0032502; GO:0040011; GO:0043473; GO:0048511; GO:0050896; GO:0051704; GO:0065007
ATF1	CEBPB	GO:0002376; GO:0008152; GO:0009987; GO:0022610; GO:0032501; GO:0032502; GO:0040007; GO:0040011; GO:0050896; GO:0051704; GO:0065007
ATF1	CEBPD	GO:0002376; GO:0022610; GO:0032501; GO:0032502; GO:0040007; GO:0050896; GO:0051234; GO:0051704; GO:0065007
ATF1	CREB1	GO:0002376; GO:0008152; GO:0009987; GO:0022414; GO:0032501; GO:0032502; GO:0040007; GO:0040011; GO:0050896; GO:0051234; GO:0051704; GO:0065007
ATF1	E2F1	GO:0002376; GO:0008152; GO:0009987; GO:0032501; GO:0032502; GO:0040011; GO:0050896; GO:0065007

ATF1	EGR1	GO:0002376; GO:0009987; GO:0022414; GO:0022610; GO:0032501; GO:0032502; GO:0043473; GO:0050896; GO:0051704; GO:0065007
ATF1	ETS1	GO:0002376; GO:0032501; GO:0032502; GO:0043473; GO:0050896; GO:0051704; GO:0065007
ATF1	NFKB1	GO:0002376; GO:0008152; GO:0009987; GO:0032501; GO:0032502; GO:0040011; GO:0043473; GO:0050896; GO:0051704; GO:0065007
ATF1	RARA	GO:0002376; GO:0009987; GO:0032502; GO:0043473; GO:0050896; GO:0051704; GO:0065007
ATF1	REL	GO:0002376; GO:0009987; GO:0032502; GO:0040007; GO:0040011; GO:0050896; GO:0051704; GO:0065007
ATF1	RELA	GO:0002376; GO:0009987; GO:0022610; GO:0032501; GO:0032502; GO:0040011; GO:0043473; GO:0048511; GO:0050896; GO:0051234; GO:0051704; GO:0065007
ATF1	SP1	GO:0002376; GO:0008152; GO:0009987; GO:0032501; GO:0032502; GO:0040011; GO:0050896; GO:0051704; GO:0065007
ATF1	SPI1	GO:0002376; GO:0022610; GO:0032501; GO:0032502; GO:0040011; GO:0050896; GO:0051704; GO:0065007
ATF1	STAT3	GO:0002376; GO:0009987; GO:0032502; GO:0043473; GO:0050896; GO:0051704; GO:0065007
ATF1	STAT5A	GO:0002376; GO:0032502; GO:0043473; GO:0050896; GO:0051704
ATF1	TP53	GO:0002376; GO:0009987; GO:0032502; GO:0043473; GO:0050896; GO:0051704; GO:0065007
BRCA1	EGR1	GO:0009987; GO:0022414; GO:0040007; GO:0043473; GO:0051179; GO:0051234; GO:0051704; GO:0065007
BRCA1	RELA	GO:0022414; GO:0032502; GO:0040007; GO:0043473; GO:0050896; GO:0051179; GO:0051704
CEBPA	CEBPB	GO:0000003; GO:0001906; GO:0002376; GO:0009987; GO:0022610; GO:0032501; GO:0032502; GO:0040011; GO:0048511; GO:0050896; GO:0051234; GO:0051704; GO:0065007
CEBPA	CREB1	GO:0002376; GO:0009987; GO:0022414; GO:0032501; GO:0032502; GO:0040011; GO:0050896; GO:0051704; GO:0065007
CEBPA	EGR1	GO:0002376; GO:0008152; GO:0009987; GO:0022414; GO:0032501; GO:0032502; GO:0050896; GO:0051234; GO:0051704; GO:0065007
CEBPA	ETS1	GO:0002376; GO:0009987; GO:0032502; GO:0040011; GO:0050896; GO:0051704; GO:0065007
CEBPA	ETV4	GO:0002376; GO:0008152; GO:0009987; GO:0032501; GO:0032502; GO:0040011; GO:0050896; GO:0065007
CEBPA	FOS	GO:0002376; GO:0009987; GO:0032501; GO:0032502; GO:0050896; GO:0051704; GO:0065007
CEBPA	JUN	GO:0002376; GO:0008152; GO:0009987; GO:0032501; GO:0032502; GO:0040007; GO:0040011; GO:0050896; GO:0051234; GO:0051704; GO:0065007
CEBPA	JUND	GO:0002376; GO:0009987; GO:0022414; GO:0032502; GO:0040011; GO:0050896; GO:0051704; GO:0065007
CEBPA	MYB	GO:0002376; GO:0008152; GO:0009987; GO:0022414; GO:0032501; GO:0040007; GO:0050896; GO:0051704; GO:0065007
CEBPA	NFIC	GO:0000003; GO:0002376; GO:0008152; GO:0009987; GO:0032501; GO:0032502; GO:0048511; GO:0050896; GO:0065007
CEBPA	NFKB1	GO:0002376; GO:0008152; GO:0009987; GO:0022610; GO:0032501; GO:0032502; GO:0040011; GO:0048511; GO:0050896; GO:0051234; GO:0051704; GO:0065007
CEBPA	POU2F1	GO:0001906; GO:0002376; GO:0009987; GO:0032502; GO:0040011; GO:0050896; GO:0051704; GO:0065007
CEBPA	RARA	GO:0001906; GO:0002376; GO:0009987; GO:0032502; GO:0040011; GO:0043473; GO:0050896; GO:0051234; GO:0051704; GO:0065007
CEBPA	RARG	GO:0002376; GO:0009987; GO:0032502; GO:0043473; GO:0050896; GO:0051704; GO:0065007
CEBPA	REL	GO:0002376; GO:0022610; GO:0032502; GO:0040011; GO:0050896;

		GO:0051704; GO:0065007
CEBPA	RELA	GO:0000003; GO:0002376; GO:0009987; GO:0022610; GO:0032501; GO:0032502; GO:0040011; GO:0048511; GO:0050896; GO:0051704; GO:0065007
CEBPA	SP1	GO:0001906; GO:0002376; GO:0008152; GO:0009987; GO:0022414; GO:0022610; GO:0032501; GO:0032502; GO:0048511; GO:0050896; GO:0051234; GO:0051704; GO:0065007
CEBPA	SPI1	GO:0002376; GO:0009987; GO:0022610; GO:0032502; GO:0040011; GO:0050896; GO:0051234; GO:0051704; GO:0065007
CEBPA	STAT3	GO:0002376; GO:0009987; GO:0032502; GO:0043473; GO:0050896; GO:0051704; GO:0065007
CEBPA	TP53	GO:0000003; GO:0002376; GO:0008152; GO:0009987; GO:0022414; GO:0032501; GO:0032502; GO:0040011; GO:0050896; GO:0051704; GO:0065007
CEBPA	USF1	GO:0001906; GO:0002376; GO:0008152; GO:0009987; GO:0022414; GO:0032501; GO:0032502; GO:0050896; GO:0051234; GO:0051704; GO:0065007
CEBPA	USF2	GO:0001906; GO:0008152; GO:0009987; GO:0022414; GO:0050896; GO:0051234; GO:0051704; GO:0065007
CEBPA	WT1	GO:0002376; GO:0022414; GO:0032501; GO:0032502; GO:0043473; GO:0048511; GO:0050896; GO:0051179; GO:0051704; GO:0065007
CEBPB	CEBPD	GO:0002376; GO:0009987; GO:0022610; GO:0032501; GO:0032502; GO:0040011; GO:0050896; GO:0051234; GO:0051704; GO:0065007
CEBPB	CREB1	GO:0002376; GO:0008152; GO:0009987; GO:0022610; GO:0032501; GO:0032502; GO:0040011; GO:0050896; GO:0051234; GO:0051704; GO:0065007
CEBPB	EGR1	GO:0002376; GO:0008152; GO:0009987; GO:0022414; GO:0032501; GO:0032502; GO:0048511; GO:0050896; GO:0051234; GO:0051704; GO:0065007
CEBPB	ETS1	GO:0002376; GO:0009987; GO:0032502; GO:0040011; GO:0050896; GO:0051704; GO:0065007
CEBPB	ETV4	GO:0002376; GO:0008152; GO:0009987; GO:0032501; GO:0032502; GO:0040007; GO:0040011; GO:0050896; GO:0051234; GO:0065007
CEBPB	FOS	GO:0002376; GO:0009987; GO:0022610; GO:0032501; GO:0032502; GO:0040007; GO:0050896; GO:0051704; GO:0065007
CEBPB	JUN	GO:0002376; GO:0008152; GO:0009987; GO:0022610; GO:0032501; GO:0032502; GO:0040011; GO:0050896; GO:0051704; GO:0065007
CEBPB	JUND	GO:0002376; GO:0032501; GO:0032502; GO:0040011; GO:0050896; GO:0051234; GO:0051704; GO:0065007
CEBPB	MYC	GO:0002376; GO:0008152; GO:0009987; GO:0022414; GO:0032502; GO:0048511; GO:0050896; GO:0051704; GO:0065007
CEBPB	NFIC	GO:0000003; GO:0002376; GO:0008152; GO:0009987; GO:0022414; GO:0032501; GO:0032502; GO:0048511; GO:0050896; GO:0051704; GO:0065007
CEBPB	NFKB1	GO:0002376; GO:0008152; GO:0009987; GO:0022610; GO:0032501; GO:0032502; GO:0040011; GO:0050896; GO:0051704; GO:0065007
CEBPB	POU2F1	GO:0001906; GO:0002376; GO:0009987; GO:0032502; GO:0040011; GO:0050896; GO:0051704; GO:0065007
CEBPB	RARA	GO:0001906; GO:0002376; GO:0009987; GO:0032502; GO:0050896; GO:0051234; GO:0051704; GO:0065007
CEBPB	REL	GO:0002376; GO:0009987; GO:0022610; GO:0032502; GO:0040011; GO:0050896; GO:0051704; GO:0065007
CEBPB	RELA	GO:0000003; GO:0002376; GO:0009987; GO:0022610; GO:0032501; GO:0032502; GO:0040011; GO:0050896; GO:0051704; GO:0065007
CEBPB	SP1	GO:0001906; GO:0002376; GO:0008152; GO:0009987; GO:0022414; GO:0032501; GO:0032502; GO:0040011; GO:0050896; GO:0051234; GO:0051704; GO:0065007
CEBPB	SPI1	GO:0002376; GO:0009987; GO:0022610; GO:0032502; GO:0040011;

		GO:0050896; GO:0051704; GO:0065007
CEBPB	TFAP2A	GO:0002376; GO:0008152; GO:0009987; GO:0022414; GO:0032501; GO:0032502; GO:0040011; GO:0050896; GO:0065007
CEBPB	TP53	GO:0000003; GO:0002376; GO:0008152; GO:0009987; GO:0032501; GO:0032502; GO:0040011; GO:0050896; GO:0051234; GO:0051704; GO:0065007
CEBPB	USF1	GO:0001906; GO:0002376; GO:0009987; GO:0032502; GO:0040007; GO:0050896; GO:0051704; GO:0065007
CEBPB	USF2	GO:0001906; GO:0002376; GO:0009987; GO:0032502; GO:0040007; GO:0050896; GO:0051704; GO:0065007
CEBPD	JUN	GO:0002376; GO:0009987; GO:0022610; GO:0032501; GO:0032502; GO:0040011; GO:0050896; GO:0051704; GO:0065007
CEBPD	NFKB1	GO:0002376; GO:0022610; GO:0032501; GO:0032502; GO:0050896; GO:0051704; GO:0065007
CEBPD	REL	GO:0002376; GO:0040007; GO:0050896; GO:0051704; GO:0065007
CEBPD	RELA	GO:0002376; GO:0022610; GO:0032501; GO:0032502; GO:0050896; GO:0051704; GO:0065007
CREB1	E2F1	GO:0002376; GO:0009987; GO:0032502; GO:0040011; GO:0050896; GO:0051704; GO:0065007
CREB1	EGR1	GO:0002376; GO:0009987; GO:0022414; GO:0022610; GO:0032501; GO:0032502; GO:0043473; GO:0050896; GO:0051704; GO:0065007
CREB1	ETS1	GO:0002376; GO:0008152; GO:0009987; GO:0022414; GO:0032501; GO:0032502; GO:0040007; GO:0040011; GO:0050896; GO:0051234; GO:0051704; GO:0065007
CREB1	JUN	GO:0002376; GO:0009987; GO:0022610; GO:0032501; GO:0032502; GO:0050896; GO:0051234; GO:0051704; GO:0065007
CREB1	MYC	GO:0000003; GO:0002376; GO:0008152; GO:0009987; GO:0022414; GO:0032502; GO:0040007; GO:0040011; GO:0050896; GO:0051704; GO:0065007
CREB1	NFKB1	GO:0002376; GO:0008152; GO:0009987; GO:0022610; GO:0032501; GO:0032502; GO:0040011; GO:0050896; GO:0051234; GO:0051704; GO:0065007
CREB1	RARA	GO:0002376; GO:0008152; GO:0009987; GO:0032501; GO:0032502; GO:0043473; GO:0050896; GO:0051704; GO:0065007
CREB1	REL	GO:0002376; GO:0009987; GO:0022610; GO:0032502; GO:0040007; GO:0040011; GO:0050896; GO:0051704; GO:0065007
CREB1	RELA	GO:0002376; GO:0009987; GO:0022610; GO:0032501; GO:0032502; GO:0043473; GO:0050896; GO:0051704; GO:0065007
CREB1	SP1	GO:0002376; GO:0008152; GO:0009987; GO:0022414; GO:0032501; GO:0032502; GO:0040007; GO:0050896; GO:0051704; GO:0065007
CREB1	SPI1	GO:0002376; GO:0009987; GO:0022610; GO:0032501; GO:0032502; GO:0050896; GO:0051704; GO:0065007
CREB1	STAT3	GO:0002376; GO:0009987; GO:0032501; GO:0032502; GO:0043473; GO:0050896; GO:0051704; GO:0065007
CREB1	STAT5A	GO:0002376; GO:0032502; GO:0043473; GO:0050896; GO:0051704; GO:0065007
CREB1	TP53	GO:0002376; GO:0008152; GO:0009987; GO:0022414; GO:0032501; GO:0032502; GO:0040007; GO:0043473; GO:0050896; GO:0051704; GO:0065007
CREB1	USF1	GO:0002376; GO:0009987; GO:0032501; GO:0032502; GO:0040011; GO:0050896; GO:0051234; GO:0051704; GO:0065007
E2F1	NFKB1	GO:0002376; GO:0008152; GO:0009987; GO:0032501; GO:0032502; GO:0040011; GO:0050896; GO:0065007
E2F4	TP53	GO:0008152; GO:0009987; GO:0022414; GO:0032502; GO:0050896; GO:0051179; GO:0065007
EGR1	ETS1	GO:0002376; GO:0009987; GO:0032501; GO:0032502; GO:0040011; GO:0043473; GO:0050896; GO:0051234; GO:0051704; GO:0065007
EGR1	JUN	GO:0002376; GO:0009987; GO:0022610; GO:0032501; GO:0032502;

		GO:0050896; GO:0051234; GO:0051704; GO:0065007
EGR1	MYC	GO:0002376; GO:0008152; GO:0009987; GO:0022414; GO:0032501; GO:0032502; GO:0050896; GO:0051179; GO:0051234; GO:0051704; GO:0065007
EGR1	NFKB1	GO:0002376; GO:0009987; GO:0022414; GO:0032501; GO:0032502; GO:0040011; GO:0050896; GO:0051234; GO:0051704; GO:0065007
EGR1	RARA	GO:0002376; GO:0009987; GO:0022414; GO:0032501; GO:0032502; GO:0043473; GO:0050896; GO:0051234; GO:0051704; GO:0065007
EGR1	RELA	GO:0002376; GO:0009987; GO:0022414; GO:0022610; GO:0032502; GO:0043473; GO:0050896; GO:0051179; GO:0051704; GO:0065007
EGR1	SP1	GO:0002376; GO:0009987; GO:0022414; GO:0022610; GO:0032501; GO:0032502; GO:0048511; GO:0050896; GO:0051179; GO:0051704; GO:0065007
EGR1	STAT3	GO:0002376; GO:0009987; GO:0032501; GO:0032502; GO:0043473; GO:0050896; GO:0051704; GO:0065007
EGR1	STAT5A	GO:0002376; GO:0009987; GO:0022610; GO:0032501; GO:0032502; GO:0043473; GO:0050896; GO:0051179; GO:0051704; GO:0065007
EGR1	TP53	GO:0002376; GO:0009987; GO:0022414; GO:0022610; GO:0032501; GO:0032502; GO:0050896; GO:0051179; GO:0051704; GO:0065007
EGR1	WT1	GO:0008152; GO:0009987; GO:0022414; GO:0032501; GO:0032502; GO:0043473; GO:0050896; GO:0051179; GO:0051704; GO:0065007
ELK1	SP1	GO:0002376; GO:0009987; GO:0022610; GO:0032501; GO:0032502; GO:0040007; GO:0048511; GO:0050896; GO:0065007
ELK1	TP53	GO:0002376; GO:0008152; GO:0009987; GO:0022610; GO:0032502; GO:0040007; GO:0040011; GO:0050896; GO:0051704; GO:0065007
EPAS1	ETS1	GO:0022414; GO:0032501; GO:0032502; GO:0048511; GO:0051704; GO:0065007
ESR1	RELA	GO:0002376; GO:0008152; GO:0009987; GO:0022610; GO:0032502; GO:0043473; GO:0048511; GO:0050896; GO:0065007
ESR1	TFAP2A	GO:0009987; GO:0022414; GO:0022610; GO:0032502; GO:0043473; GO:0050896; GO:0051704; GO:0065007
ESR1	TP53	GO:0008152; GO:0009987; GO:0032502; GO:0043473; GO:0050896; GO:0051179; GO:0051704; GO:0065007
ETS1	ETS2	GO:0000003; GO:0002376; GO:0008152; GO:0009987; GO:0022414; GO:0022610; GO:0032501; GO:0032502; GO:0040011; GO:0050896; GO:0051704; GO:0065007
ETS1	JUN	GO:0002376; GO:0009987; GO:0022610; GO:0032501; GO:0032502; GO:0050896; GO:0051704; GO:0065007
ETS1	MYB	GO:0000003; GO:0002376; GO:0009987; GO:0032501; GO:0032502; GO:0043473; GO:0050896; GO:0051704; GO:0065007
ETS1	MYC	GO:0002376; GO:0008152; GO:0009987; GO:0032502; GO:0043473; GO:0050896; GO:0051704; GO:0065007
ETS1	NFKB1	GO:0002376; GO:0008152; GO:0009987; GO:0022414; GO:0022610; GO:0032501; GO:0032502; GO:0040011; GO:0043473; GO:0050896; GO:0051704; GO:0065007
ETS1	POU2F1	GO:0002376; GO:0009987; GO:0022610; GO:0032502; GO:0050896; GO:0051704; GO:0065007
ETS1	PPARG	GO:0009987; GO:0022414; GO:0032501; GO:0032502; GO:0043473; GO:0050896; GO:0051704; GO:0065007
ETS1	RARA	GO:0002376; GO:0009987; GO:0022414; GO:0032501; GO:0032502; GO:0043473; GO:0050896; GO:0051704; GO:0065007
ETS1	RARB	GO:0002376; GO:0009987; GO:0022414; GO:0032501; GO:0032502; GO:0040007; GO:0043473; GO:0050896; GO:0051704; GO:0065007
ETS1	RELA	GO:0002376; GO:0008152; GO:0009987; GO:0032501; GO:0032502; GO:0043473; GO:0050896; GO:0051704; GO:0065007
ETS1	SP1	GO:0002376; GO:0008152; GO:0009987; GO:0032501; GO:0032502; GO:0040007; GO:0040011; GO:0043473; GO:0050896; GO:0051704; GO:0065007

ETS1	SPI1	GO:0002376; GO:0009987; GO:0022610; GO:0032501; GO:0032502; GO:0050896; GO:0051704; GO:0065007
ETS1	TP53	GO:0002376; GO:0008152; GO:0009987; GO:0022414; GO:0032501; GO:0032502; GO:0040007; GO:0043473; GO:0050896; GO:0051704; GO:0065007
ETS1	USF1	GO:0002376; GO:0032501; GO:0032502; GO:0040011; GO:0050896; GO:0051234; GO:0051704; GO:0065007
ETS2	POU2F1	GO:0002376; GO:0009987; GO:0022610; GO:0032502; GO:0043473; GO:0050896; GO:0065007
ETS2	SP1	GO:0002376; GO:0008152; GO:0009987; GO:0032501; GO:0032502; GO:0040011; GO:0050896; GO:0051704; GO:0065007
ETS2	TP53	GO:0002376; GO:0008152; GO:0009987; GO:0032502; GO:0043473; GO:0050896; GO:0051704; GO:0065007
ETV4	NFKB1	GO:0002376; GO:0008152; GO:0009987; GO:0032501; GO:0032502; GO:0040011; GO:0050896; GO:0051704; GO:0065007
ETV4	POU2F1	GO:0002376; GO:0009987; GO:0022610; GO:0032502; GO:0040011; GO:0050896; GO:0051704; GO:0065007
ETV4	TFAP2A	GO:0002376; GO:0008152; GO:0009987; GO:0032501; GO:0032502; GO:0040011; GO:0050896; GO:0051234; GO:0065007
ETV4	TP53	GO:0002376; GO:0008152; GO:0009987; GO:0032501; GO:0032502; GO:0040011; GO:0050896; GO:0065007
FOS	JUN	GO:0002376; GO:0008152; GO:0009987; GO:0022414; GO:0032501; GO:0032502; GO:0040007; GO:0040011; GO:0048511; GO:0050896; GO:0051704; GO:0065007
FOS	NFKB1	GO:0002376; GO:0008152; GO:0009987; GO:0032501; GO:0032502; GO:0040011; GO:0050896; GO:0065007
FOS	REL	GO:0002376; GO:0009987; GO:0022610; GO:0032502; GO:0040007; GO:0050896; GO:0065007
FOS	RELA	GO:0002376; GO:0008152; GO:0009987; GO:0022610; GO:0032501; GO:0032502; GO:0048511; GO:0050896; GO:0051704; GO:0065007
FOS	SP1	GO:0002376; GO:0008152; GO:0009987; GO:0032501; GO:0032502; GO:0050896; GO:0065007
JUN	JUND	GO:0002376; GO:0032501; GO:0040011; GO:0050896; GO:0051704; GO:0065007
JUN	LEF1	GO:0002376; GO:0009987; GO:0040011; GO:0043473; GO:0050896; GO:0051704
JUN	NFKB1	GO:0002376; GO:0008152; GO:0009987; GO:0022610; GO:0032501; GO:0032502; GO:0040011; GO:0050896; GO:0051704; GO:0065007
JUN	POU2F2	GO:0002376; GO:0009987; GO:0022610; GO:0032502; GO:0043473; GO:0050896; GO:0065007
JUN	REL	GO:0002376; GO:0009987; GO:0022610; GO:0032502; GO:0040011; GO:0050896; GO:0051704; GO:0065007
JUN	RELA	GO:0002376; GO:0009987; GO:0022414; GO:0022610; GO:0032501; GO:0032502; GO:0040011; GO:0050896; GO:0051704; GO:0065007
JUN	SP1	GO:0002376; GO:0008152; GO:0009987; GO:0022414; GO:0022610; GO:0032501; GO:0032502; GO:0040011; GO:0048511; GO:0050896; GO:0051704; GO:0065007
JUN	SPI1	GO:0002376; GO:0009987; GO:0022610; GO:0032502; GO:0050896; GO:0051704; GO:0065007
JUN	TP53	GO:0002376; GO:0008152; GO:0009987; GO:0032501; GO:0032502; GO:0050896; GO:0051704; GO:0065007
JUND	NFKB1	GO:0002376; GO:0022610; GO:0050896; GO:0051704; GO:0065007
MYB	PPARG	GO:0009987; GO:0032502; GO:0040007; GO:0043473; GO:0050896; GO:0051179; GO:0065007
MYB	RARB	GO:0002376; GO:0009987; GO:0032502; GO:0040007; GO:0043473; GO:0050896; GO:0051704; GO:0065007
MYB	RELA	GO:0022414; GO:0032501; GO:0032502; GO:0043473; GO:0050896; GO:0065007

MYB	SP1	GO:0002376; GO:0008152; GO:0009987; GO:0022414; GO:0032502; GO:0043473; GO:0050896; GO:0051704; GO:0065007
MYB	STAT3	GO:0002376; GO:0009987; GO:0032502; GO:0040007; GO:0043473; GO:0050896; GO:0065007
MYB	TP53	GO:0002376; GO:0008152; GO:0009987; GO:0022414; GO:0032502; GO:0043473; GO:0050896; GO:0065007
MYBL2	TFAP2A	GO:0002376; GO:0008152; GO:0022414; GO:0032502; GO:0043473; GO:0050896; GO:0051704; GO:0065007
MYC	NFKB1	GO:0002376; GO:0008152; GO:0009987; GO:0022414; GO:0032502; GO:0043473; GO:0050896; GO:0051704; GO:0065007
MYC	PPARG	GO:0002376; GO:0009987; GO:0022414; GO:0032502; GO:0043473; GO:0051704; GO:0065007
MYC	RELA	GO:0002376; GO:0008152; GO:0009987; GO:0022414; GO:0032502; GO:0043473; GO:0050896; GO:0051704; GO:0065007
MYC	SP1	GO:0002376; GO:0008152; GO:0009987; GO:0022414; GO:0032502; GO:0043473; GO:0050896; GO:0051704; GO:0065007
MYC	TP53	GO:0002376; GO:0008152; GO:0009987; GO:0022414; GO:0032502; GO:0043473; GO:0050896; GO:0051179; GO:0051704; GO:0065007
NFIC	RELA	GO:0000003; GO:0008152; GO:0009987; GO:0022414; GO:0032501; GO:0032502; GO:0043473; GO:0048511; GO:0050896; GO:0051704; GO:0065007
NFIC	SP1	GO:0008152; GO:0009987; GO:0022414; GO:0032501; GO:0032502; GO:0040011; GO:0048511; GO:0050896; GO:0065007
NFIC	TP53	GO:0000003; GO:0008152; GO:0009987; GO:0032501; GO:0032502; GO:0043473; GO:0050896; GO:0065007
NFKB1	NFKB2	GO:0002376; GO:0009987; GO:0022610; GO:0032502; GO:0043473; GO:0050896; GO:0051704; GO:0065007
NFKB1	POU2F1	GO:0002376; GO:0008152; GO:0009987; GO:0022610; GO:0032501; GO:0032502; GO:0040011; GO:0050896; GO:0051704; GO:0065007
NFKB1	POU2F2	GO:0002376; GO:0009987; GO:0032502; GO:0043473; GO:0050896; GO:0065007
NFKB1	PPARG	GO:0002376; GO:0009987; GO:0032501; GO:0032502; GO:0040007; GO:0043473; GO:0050896; GO:0051704; GO:0065007
NFKB1	RARA	GO:0002376; GO:0009987; GO:0022414; GO:0032502; GO:0040011; GO:0043473; GO:0050896; GO:0051704; GO:0065007
NFKB1	RARB	GO:0002376; GO:0008152; GO:0009987; GO:0022414; GO:0032502; GO:0043473; GO:0050896; GO:0051704; GO:0065007
NFKB1	RARG	GO:0002376; GO:0009987; GO:0022414; GO:0032502; GO:0043473; GO:0050896; GO:0051704; GO:0065007
NFKB1	REL	GO:0002376; GO:0009987; GO:0022610; GO:0032502; GO:0040011; GO:0050896; GO:0051704; GO:0065007
NFKB1	RELA	GO:0002376; GO:0009987; GO:0022414; GO:0022610; GO:0032501; GO:0032502; GO:0040007; GO:0040011; GO:0043473; GO:0050896; GO:0051704; GO:0065007
NFKB1	SP1	GO:0002376; GO:0008152; GO:0009987; GO:0022414; GO:0022610; GO:0032501; GO:0032502; GO:0040007; GO:0040011; GO:0043473; GO:0048511; GO:0050896; GO:0051704; GO:0065007
NFKB1	SPI1	GO:0002376; GO:0022610; GO:0032502; GO:0040011; GO:0050896; GO:0051234; GO:0051704; GO:0065007
NFKB1	STAT1	GO:0002376; GO:0008152; GO:0009987; GO:0032502; GO:0040011; GO:0050896; GO:0051704; GO:0065007
NFKB1	STAT3	GO:0002376; GO:0008152; GO:0009987; GO:0032502; GO:0040011; GO:0043473; GO:0050896; GO:0051704; GO:0065007
NFKB1	STAT5A	GO:0002376; GO:0009987; GO:0032502; GO:0043473; GO:0050896; GO:0051704; GO:0065007
NFKB1	STAT6	GO:0002376; GO:0009987; GO:0022610; GO:0032502; GO:0040011; GO:0050896; GO:0051704; GO:0065007
NFKB1	TFAP2A	GO:0002376; GO:0008152; GO:0009987; GO:0032501; GO:0032502; GO:0040011; GO:0050896; GO:0065007

NFKB1	TP53	GO:0002376; GO:0008152; GO:0009987; GO:0032501; GO:0032502; GO:0040007; GO:0040011; GO:0043473; GO:0050896; GO:0051704; GO:0065007
NFKB1	WT1	GO:0002376; GO:0009987; GO:0022414; GO:0032501; GO:0032502; GO:0043473; GO:0048511; GO:0050896; GO:0051179; GO:0051234; GO:0051704; GO:0065007
NFKB2	RELA	GO:0002376; GO:0009987; GO:0022610; GO:0032502; GO:0043473; GO:0050896; GO:0051179; GO:0051704; GO:0065007
POU2F1	POU2F2	GO:0002376; GO:0009987; GO:0032502; GO:0040011; GO:0043473; GO:0050896; GO:0051704; GO:0065007
POU2F1	RARA	GO:0001906; GO:0002376; GO:0009987; GO:0032502; GO:0040011; GO:0050896; GO:0065007
POU2F1	RELA	GO:0002376; GO:0009987; GO:0032502; GO:0040011; GO:0043473; GO:0050896; GO:0051704; GO:0065007
POU2F1	SP1	GO:0001906; GO:0002376; GO:0009987; GO:0022610; GO:0032501; GO:0032502; GO:0050896; GO:0051704; GO:0065007
POU2F1	SPI1	GO:0002376; GO:0022610; GO:0032501; GO:0032502; GO:0050896; GO:0051704; GO:0065007
POU2F1	TFAP2A	GO:0002376; GO:0009987; GO:0032502; GO:0040011; GO:0043473; GO:0050896; GO:0051704; GO:0065007
POU2F1	TP53	GO:0002376; GO:0008152; GO:0009987; GO:0032502; GO:0040011; GO:0043473; GO:0050896; GO:0065007
POU2F1	USF1	GO:0001906; GO:0009987; GO:0022414; GO:0032502; GO:0043473; GO:0050896; GO:0051704; GO:0065007
POU2F1	USF2	GO:0001906; GO:0002376; GO:0009987; GO:0022414; GO:0032502; GO:0043473; GO:0050896; GO:0051704; GO:0065007
POU2F2	RELA	GO:0002376; GO:0009987; GO:0032502; GO:0040011; GO:0043473; GO:0050896; GO:0065007
PPARG	RARB	GO:0008152; GO:0009987; GO:0022414; GO:0032501; GO:0032502; GO:0040007; GO:0043473; GO:0050896; GO:0051704; GO:0065007
PPARG	RELA	GO:0002376; GO:0008152; GO:0009987; GO:0032501; GO:0032502; GO:0040007; GO:0043473; GO:0050896; GO:0051704; GO:0065007
PPARG	SP1	GO:0002376; GO:0008152; GO:0009987; GO:0022414; GO:0032501; GO:0032502; GO:0043473; GO:0050896; GO:0051704; GO:0065007
PPARG	STAT3	GO:0002376; GO:0009987; GO:0032502; GO:0043473; GO:0050896; GO:0051704; GO:0065007
PPARG	STAT5A	GO:0002376; GO:0009987; GO:0032502; GO:0043473; GO:0050896; GO:0051179; GO:0051704; GO:0065007
PPARG	TP53	GO:0002376; GO:0008152; GO:0009987; GO:0022414; GO:0032501; GO:0032502; GO:0040007; GO:0043473; GO:0050896; GO:0051704; GO:0065007
RARA	REL	GO:0002376; GO:0009987; GO:0032502; GO:0050896; GO:0051704; GO:0065007
RARA	RELA	GO:0002376; GO:0009987; GO:0022414; GO:0032502; GO:0043473; GO:0050896; GO:0051704; GO:0065007
RARA	SP1	GO:0001906; GO:0002376; GO:0008152; GO:0009987; GO:0022414; GO:0032501; GO:0032502; GO:0040011; GO:0050896; GO:0051704; GO:0065007
RARA	TP53	GO:0002376; GO:0009987; GO:0022414; GO:0032502; GO:0040011; GO:0043473; GO:0050896; GO:0051704; GO:0065007
RARB	RELA	GO:0002376; GO:0008152; GO:0009987; GO:0032502; GO:0043473; GO:0050896; GO:0051704; GO:0065007
RARB	SP1	GO:0009987; GO:0022414; GO:0032502; GO:0043473; GO:0050896; GO:0051704; GO:0065007
RARB	TP53	GO:0002376; GO:0008152; GO:0009987; GO:0022414; GO:0032502; GO:0040011; GO:0043473; GO:0050896; GO:0051704; GO:0065007
RARG	RELA	GO:0002376; GO:0032502; GO:0043473; GO:0050896; GO:0051704; GO:0065007

RARG	TP53	GO:0002376; GO:0009987; GO:0022414; GO:0032502; GO:0040011; GO:0043473; GO:0050896; GO:0051704; GO:0065007
REL	RELA	GO:0002376; GO:0009987; GO:0022610; GO:0032502; GO:0040011; GO:0050896; GO:0051704; GO:0065007
REL	SP1	GO:0002376; GO:0009987; GO:0022610; GO:0032502; GO:0040007; GO:0040011; GO:0050896; GO:0051179; GO:0065007
REL	SPI1	GO:0002376; GO:0009987; GO:0022610; GO:0032502; GO:0040011; GO:0050896; GO:0051704; GO:0065007
REL	STAT1	GO:0002376; GO:0009987; GO:0022610; GO:0032502; GO:0040011; GO:0050896; GO:0051179; GO:0051704; GO:0065007
REL	STAT5A	GO:0002376; GO:0009987; GO:0032502; GO:0050896; GO:0051179; GO:0051704; GO:0065007
REL	TP53	GO:0002376; GO:0009987; GO:0032502; GO:0040007; GO:0040011; GO:0050896; GO:0051179; GO:0065007
RELA	SMAD4	GO:0022414; GO:0022610; GO:0032502; GO:0040007; GO:0050896
RELA	SP1	GO:0002376; GO:0008152; GO:0009987; GO:0022414; GO:0022610; GO:0032501; GO:0032502; GO:0040007; GO:0040011; GO:0043473; GO:0048511; GO:0050896; GO:0051704; GO:0065007
RELA	SPI1	GO:0002376; GO:0009987; GO:0022610; GO:0032502; GO:0040011; GO:0050896; GO:0051704; GO:0065007
RELA	STAT1	GO:0002376; GO:0008152; GO:0009987; GO:0022414; GO:0022610; GO:0032502; GO:0040011; GO:0043473; GO:0050896; GO:0051179; GO:0051704; GO:0065007
RELA	STAT3	GO:0002376; GO:0009987; GO:0022414; GO:0032502; GO:0043473; GO:0050896; GO:0051704; GO:0065007
RELA	STAT5A	GO:0002376; GO:0009987; GO:0022610; GO:0032502; GO:0043473; GO:0050896; GO:0051179; GO:0051704; GO:0065007
RELA	TFAP2A	GO:0002376; GO:0008152; GO:0009987; GO:0022610; GO:0032502; GO:0040011; GO:0043473; GO:0050896; GO:0065007
RELA	TP53	GO:0000003; GO:0002376; GO:0008152; GO:0009987; GO:0022414; GO:0022610; GO:0032501; GO:0032502; GO:0040007; GO:0040011; GO:0043473; GO:0050896; GO:0051179; GO:0051704; GO:0065007
RELA	WT1	GO:0008152; GO:0009987; GO:0022414; GO:0032502; GO:0043473; GO:0050896; GO:0051179; GO:0051704; GO:0065007
SMAD3	SP1	GO:0008152; GO:0009987; GO:0022414; GO:0032501; GO:0032502; GO:0043473; GO:0050896; GO:0065007
SP1	SPI1	GO:0002376; GO:0008152; GO:0009987; GO:0022610; GO:0032501; GO:0032502; GO:0050896; GO:0051704; GO:0065007
SP1	STAT3	GO:0002376; GO:0008152; GO:0009987; GO:0022414; GO:0022610; GO:0032502; GO:0040011; GO:0043473; GO:0050896; GO:0051704; GO:0065007
SP1	TFAP2A	GO:0002376; GO:0008152; GO:0009987; GO:0022414; GO:0022610; GO:0032501; GO:0032502; GO:0040007; GO:0040011; GO:0043473; GO:0050896; GO:0051704; GO:0065007
SP1	TP53	GO:0002376; GO:0008152; GO:0009987; GO:0022414; GO:0032501; GO:0032502; GO:0040007; GO:0040011; GO:0043473; GO:0050896; GO:0051179; GO:0051704; GO:0065007
SP1	WT1	GO:0002376; GO:0008152; GO:0009987; GO:0022414; GO:0032502; GO:0043473; GO:0050896; GO:0065007
STAT3	STAT5A	GO:0002376; GO:0009987; GO:0032501; GO:0032502; GO:0043473; GO:0050896; GO:0051704; GO:0065007
STAT3	TP53	GO:0002376; GO:0009987; GO:0032501; GO:0032502; GO:0043473; GO:0050896; GO:0051704; GO:0065007
STAT5A	TP53	GO:0002376; GO:0009987; GO:0022610; GO:0032501; GO:0032502; GO:0043473; GO:0050896; GO:0051179; GO:0051704; GO:0065007
TFAP2A	TP53	GO:0002376; GO:0008152; GO:0009987; GO:0032502; GO:0040011; GO:0050896; GO:0065007
USF1	USF2	GO:0001906; GO:0002376; GO:0008152; GO:0009987; GO:0022414;

		GO:0022610; GO:0032501; GO:0032502; GO:0040011; GO:0050896; GO:0051234; GO:0051704; GO:0065007

B. TF-miRNA Co-regulation

TF	miRNA	Enriched GO IDs
EGR1	miR-493-5p	GO:0009987; GO:0022414; GO:0032502; GO:0040007; GO:0043473; GO:0051234; GO:0065007
NFKB1	miR-30-5p	GO:0002376; GO:0009987; GO:0022414; GO:0032501; GO:0032502; GO:0043473; GO:0051704; GO:0065007
RARA	miR-30-5p	GO:0022414; GO:0032501; GO:0032502; GO:0043473; GO:0048511; GO:0065007
TP53	miR-204/211	GO:0009987; GO:0022414; GO:0032501; GO:0032502; GO:0043473; GO:0048511; GO:0050896; GO:0051179; GO:0051704; GO:0065007
ETS1	miR-503	GO:0043473; GO:0050896; GO:0051704; GO:0065007
CREB1	miR-496	GO:0009987; GO:0022414; GO:0032501; GO:0032502; GO:0043473; GO:0065007
SP1	miR-124/506	GO:0002376; GO:0008152; GO:0009987; GO:0022414; GO:0022610; GO:0032501; GO:0032502; GO:0040011; GO:0043473; GO:0050896; GO:0051234; GO:0065007
SP1	miR-182	GO:0002376; GO:0022414; GO:0032502; GO:0043473; GO:0050896; GO:0065007
CREB1	miR-27	GO:0000003; GO:0009987; GO:0032501; GO:0032502; GO:0043473; GO:0050896; GO:0065007
CREB1	miR-26	GO:0009987; GO:0032502; GO:0043473; GO:0050896; GO:0051704; GO:0065007
RARB	miR-493-5p	GO:0009987; GO:0032502; GO:0043473; GO:0050896; GO:0051704; GO:0065007
TP53	miR-369-3p	GO:0022414; GO:0032502; GO:0048511; GO:0050896; GO:0051704; GO:0065007
SP1	miR-149	GO:0032502; GO:0043473; GO:0050896; GO:0051704; GO:0065007
MYB	miR-181	GO:0000003; GO:0002376; GO:0009987; GO:0022414; GO:0032501; GO:0032502; GO:0043473; GO:0050896; GO:0051704; GO:0065007
TP53	miR-494	GO:0008152; GO:0009987; GO:0022414; GO:0032502; GO:0048511; GO:0050896; GO:0051704; GO:0065007
CREB1	miR-205	GO:0040007; GO:0043473; GO:0048511; GO:0065007
TP53	miR-34/449	GO:0002376; GO:0009987; GO:0022414; GO:0032501; GO:0032502; GO:0043473; GO:0048511; GO:0050896; GO:0051704; GO:0065007
SP1	miR-493-5p	GO:0002376; GO:0009987; GO:0022414; GO:0032502; GO:0043473; GO:0051704; GO:0065007
NFKB1	miR-503	GO:0002376; GO:0009987; GO:0032501; GO:0032502; GO:0043473; GO:0050896; GO:0065007
TP53	miR-96	GO:0002376; GO:0009987; GO:0022414; GO:0032501; GO:0032502; GO:0043473; GO:0050896; GO:0051704; GO:0065007
TP53	miR-30-5p	GO:0002376; GO:0008152; GO:0009987; GO:0022414; GO:0032501; GO:0032502; GO:0043473; GO:0048511; GO:0050896; GO:0051704; GO:0065007
CREB1	miR-96	GO:0032501; GO:0032502; GO:0043473; GO:0050896; GO:0065007
ETS1	miR-181	GO:0000003; GO:0002376; GO:0009987; GO:0022414; GO:0032501; GO:0032502; GO:0043473; GO:0050896; GO:0051704; GO:0065007
ATF1	miR-205	GO:0032502; GO:0040007; GO:0043473; GO:0048511; GO:0065007
CREB1	miR-381	GO:0009987; GO:0032501; GO:0032502; GO:0043473; GO:0050896; GO:0065007
POU2F1	miR-496	GO:0002376; GO:0022610; GO:0032501; GO:0032502; GO:0043473; GO:0050896; GO:0065007
SP1	miR-96	GO:0002376; GO:0022414; GO:0032501; GO:0032502; GO:0043473;

		GO:0050896; GO:0065007
ETS1	miR-30-5p	GO:0009987; GO:0022414; GO:0022610; GO:0032501; GO:0032502; GO:0043473; GO:0051704; GO:0065007
TP53	miR-153	GO:0002376; GO:0008152; GO:0009987; GO:0022414; GO:0032502; GO:0043473; GO:0048511; GO:0050896; GO:0051704; GO:0065007
TP53	miR-15/16/195/424/497	GO:0002376; GO:0009987; GO:0022414; GO:0032502; GO:0043473; GO:0050896; GO:0065007
SP1	miR-30-5p	GO:0002376; GO:0009987; GO:0022414; GO:0032501; GO:0032502; GO:0043473; GO:0050896; GO:0065007
SP1	miR-15/16/195/424/497	GO:0002376; GO:0008152; GO:0009987; GO:0022414; GO:0032501; GO:0032502; GO:0043473; GO:0050896; GO:0051704; GO:0065007
TP53	miR-181	GO:0002376; GO:0008152; GO:0009987; GO:0022414; GO:0032502; GO:0043473; GO:0048511; GO:0050896; GO:0051704; GO:0065007
CREB1	miR-182	GO:0032501; GO:0032502; GO:0043473; GO:0050896; GO:0065007
CREB1	miR-181	GO:0009987; GO:0022414; GO:0032501; GO:0032502; GO:0043473; GO:0051704; GO:0065007
NFKB1	miR-143	GO:0002376; GO:0009987; GO:0032502; GO:0040007; GO:0043473; GO:0050896; GO:0065007
MYB	miR-30-5p	GO:0002376; GO:0009987; GO:0022414; GO:0032502; GO:0043473; GO:0050896; GO:0051704; GO:0065007
AR	miR-15/16/195/424/497	GO:0009987; GO:0022414; GO:0032501; GO:0032502; GO:0043473; GO:0050896; GO:0051704; GO:0065007
NFKB1	miR-493-5p	GO:0002376; GO:0009987; GO:0022414; GO:0032502; GO:0040007; GO:0043473; GO:0050896; GO:0051704; GO:0065007
RARA	miR-493-5p	GO:0022414; GO:0032502; GO:0040007; GO:0043473; GO:0065007
ETS2	miR-181	GO:0000003; GO:0002376; GO:0022414; GO:0032501; GO:0032502; GO:0050896; GO:0051704; GO:0065007

C. miRNA-miRNA Co-regulation

**miRNA A**	**miRNA B**	**Enriched GO ID**
let-7/98	miR-410	GO:0009987; GO:0032501; GO:0032502; GO:0040007; GO:0051179; GO:0065007
let-7/98	miR-493-5p	GO:0008152; GO:0009987; GO:0032502; GO:0040007; GO:0051179; GO:0065007
miR-101	miR-124/506	GO:0008152; GO:0009987; GO:0022610; GO:0032501; GO:0032502; GO:0065007
miR-101	miR-144	GO:0008152; GO:0009987; GO:0022610; GO:0032501; GO:0032502; GO:0040007; GO:0065007
miR-101	miR-369-3p	GO:0008152; GO:0009987; GO:0032502; GO:0065007
miR-124.1	miR-124/506	GO:0001906; GO:0008152; GO:0009987; GO:0022610; GO:0032501; GO:0032502; GO:0040007; GO:0043473; GO:0051179; GO:0051234; GO:0065007
miR-124.1	miR-190	GO:0001906; GO:0022610; GO:0032502; GO:0043473; GO:0065007
miR-124.1	miR-30-5p	GO:0008152; GO:0009987; GO:0032501; GO:0032502; GO:0043473; GO:0051234; GO:0065007
miR-124/506	miR-144	GO:0008152; GO:0009987; GO:0032501; GO:0032502; GO:0051234; GO:0065007
miR-124/506	miR-15/16/195/424/497	GO:0008152; GO:0009987; GO:0022610; GO:0032501; GO:0032502; GO:0051234; GO:0065007
miR-124/506	miR-153	GO:0008152; GO:0009987; GO:0022610; GO:0032502; GO:0065007
miR-124/506	miR-181	GO:0008152; GO:0009987; GO:0022610; GO:0032502; GO:0051234; GO:0065007
miR-124/506	miR-182	GO:0008152; GO:0009987; GO:0032502; GO:0043473;

		GO:0051179; GO:0051234; GO:0065007
miR-124/506	miR-185	GO:0009987; GO:0022610; GO:0032502
miR-124/506	miR-186	GO:0009987; GO:0022610; GO:0032502; GO:0065007
miR-124/506	miR-19	GO:0008152; GO:0009987; GO:0022610; GO:0032502; GO:0051234; GO:0065007
miR-124/506	miR-190	GO:0001906; GO:0009987; GO:0022610; GO:0032502; GO:0065007
miR-124/506	miR-218	GO:0009987; GO:0022610; GO:0032502; GO:0051234; GO:0065007
miR-124/506	miR-221/222	GO:0009987; GO:0022610; GO:0032502; GO:0065007
miR-124/506	miR-23	GO:0008152; GO:0009987; GO:0022610; GO:0032502; GO:0065007
miR-124/506	miR-29	GO:0008152; GO:0009987; GO:0022610; GO:0032502; GO:0051234; GO:0051704; GO:0065007
miR-124/506	miR-30-5p	GO:0008152; GO:0009987; GO:0022610; GO:0032501; GO:0032502; GO:0043473; GO:0048511; GO:0051234; GO:0065007
miR-124/506	miR-320	GO:0008152; GO:0009987; GO:0022610; GO:0032502; GO:0043473; GO:0065007
miR-124/506	miR-329	GO:0001906; GO:0009987; GO:0022610; GO:0032502; GO:0065007
miR-124/506	miR-330	GO:0009987; GO:0022610; GO:0032502; GO:0065007
miR-124/506	miR-362	GO:0009987; GO:0022610; GO:0032502
miR-124/506	miR-369-3p	GO:0008152; GO:0009987; GO:0022610; GO:0032502; GO:0048511; GO:0065007
miR-124/506	miR-448	GO:0008152; GO:0009987; GO:0022610; GO:0032502; GO:0065007
miR-124/506	miR-495	GO:0008152; GO:0009987; GO:0022610; GO:0032502; GO:0065007
miR-124/506	miR-503	GO:0009987; GO:0022610; GO:0032501; GO:0032502
miR-124/506	miR-505	GO:0009987; GO:0022610; GO:0032502; GO:0065007
miR-124/506	miR-539	GO:0008152; GO:0009987; GO:0022610; GO:0032502; GO:0043473; GO:0065007
miR-124/506	miR-93.hd/291-3p/294/295/302/372/373/520	GO:0008152; GO:0009987; GO:0022610; GO:0032502; GO:0065007
miR-128	miR-27	GO:0008152; GO:0009987; GO:0022610; GO:0032501; GO:0032502; GO:0051234; GO:0065007
miR-128	miR-369-3p	GO:0008152; GO:0009987; GO:0022414; GO:0032501; GO:0032502; GO:0043473; GO:0048511; GO:0065007
miR-130/301	miR-148/152	GO:0008152; GO:0009987; GO:0032501; GO:0032502; GO:0048511; GO:0051234; GO:0065007
miR-130/301	miR-96	GO:0008152; GO:0009987; GO:0032502; GO:0048511; GO:0051234; GO:0065007
miR-133	miR-135	GO:0009987; GO:0022610; GO:0065007
miR-133	miR-153	GO:0009987; GO:0022610
miR-133	miR-448	GO:0009987; GO:0022610
miR-133	miR-7	GO:0009987; GO:0022610
miR-135	miR-153	GO:0008152; GO:0009987; GO:0022610; GO:0065007
miR-135	miR-448	GO:0009987; GO:0022610; GO:0065007
miR-135	miR-7	GO:0009987; GO:0022610
miR-138	miR-486	GO:0001906; GO:0022414; GO:0032502; GO:0040007
miR-141/200a	miR-381	GO:0008152; GO:0009987; GO:0022610; GO:0032501; GO:0032502; GO:0065007
miR-144	miR-186	GO:0008152; GO:0009987; GO:0022610; GO:0032501; GO:0032502; GO:0051704; GO:0065007
miR-144	miR-369-3p	GO:0008152; GO:0009987; GO:0032501; GO:0032502;

		GO:0065007
miR-145	miR-374	GO:0008152; GO:0009987; GO:0022610; GO:0032502; GO:0065007
miR-15/16/195/424/497	miR-153	GO:0009987; GO:0022610; GO:0032502; GO:0051234; GO:0051704; GO:0065007
miR-15/16/195/424/497	miR-185	GO:0009987; GO:0022610; GO:0032502; GO:0065007
miR-15/16/195/424/497	miR-186	GO:0008152; GO:0009987; GO:0022610; GO:0032502; GO:0051704; GO:0065007
miR-15/16/195/424/497	miR-19	GO:0008152; GO:0009987; GO:0022610; GO:0032502; GO:0065007
miR-15/16/195/424/497	miR-218	GO:0009987; GO:0022610; GO:0032502; GO:0051704; GO:0065007
miR-15/16/195/424/497	miR-221/222	GO:0009987; GO:0022610; GO:0032502; GO:0065007
miR-15/16/195/424/497	miR-23	GO:0008152; GO:0009987; GO:0022610; GO:0032502; GO:0065007
miR-15/16/195/424/497	miR-29	GO:0008152; GO:0009987; GO:0022610; GO:0032502; GO:0051704; GO:0065007
miR-15/16/195/424/497	miR-320	GO:0009987; GO:0022610; GO:0032502; GO:0065007
miR-15/16/195/424/497	miR-329	GO:0001906; GO:0009987; GO:0022610; GO:0032502
miR-15/16/195/424/497	miR-330	GO:0001906; GO:0009987; GO:0022610; GO:0032501; GO:0032502; GO:0040007; GO:0051704; GO:0065007
miR-15/16/195/424/497	miR-362	GO:0009987; GO:0022610; GO:0032502
miR-15/16/195/424/497	miR-369-3p	GO:0009987; GO:0022610; GO:0032502; GO:0065007
miR-15/16/195/424/497	miR-448	GO:0001906; GO:0008152; GO:0009987; GO:0022414; GO:0022610; GO:0032502; GO:0043473; GO:0051234; GO:0051704; GO:0065007
miR-15/16/195/424/497	miR-495	GO:0001906; GO:0008152; GO:0009987; GO:0022610; GO:0032502; GO:0065007
miR-15/16/195/424/497	miR-505	GO:0008152; GO:0009987; GO:0022610; GO:0032502
miR-15/16/195/424/497	miR-539	GO:0009987; GO:0022610; GO:0032502; GO:0065007
miR-15/16/195/424/497	miR-93.hd/291-3p/294/295/302/372/373/520	GO:0008152; GO:0009987; GO:0022414; GO:0022610; GO:0032502; GO:0065007
miR-153	miR-17-5p/20/93.mr/106/519.d	GO:0008152; GO:0009987; GO:0022610; GO:0032502; GO:0065007
miR-153	miR-181	GO:0009987; GO:0022610; GO:0032501; GO:0032502; GO:0065007
miR-153	miR-182	GO:0009987; GO:0032502; GO:0043473; GO:0051234; GO:0065007
miR-153	miR-185	GO:0009987; GO:0022610; GO:0032502; GO:0065007
miR-153	miR-186	GO:0008152; GO:0009987; GO:0022610; GO:0032502; GO:0065007
miR-153	miR-19	GO:0009987; GO:0022610; GO:0032502; GO:0065007
miR-153	miR-218	GO:0009987; GO:0022610; GO:0032502; GO:0051234; GO:0065007
miR-153	miR-221/222	GO:0009987; GO:0022610; GO:0032502; GO:0065007
miR-153	miR-23	GO:0008152; GO:0009987; GO:0022610; GO:0032502; GO:0065007
miR-153	miR-29	GO:0008152; GO:0009987; GO:0022610; GO:0032502; GO:0051704; GO:0065007

miR-153	miR-320	GO:0009987; GO:0022610; GO:0032502; GO:0065007
miR-153	miR-329	GO:0009987; GO:0022610; GO:0032502
miR-153	miR-330	GO:0009987; GO:0022610; GO:0032502
miR-153	miR-362	GO:0009987; GO:0022610; GO:0032502
miR-153	miR-369-3p	GO:0009987; GO:0022610; GO:0032502; GO:0065007
miR-153	miR-448	GO:0008152; GO:0009987; GO:0022610; GO:0032502; GO:0051234; GO:0065007
miR-153	miR-495	GO:0008152; GO:0009987; GO:0022610; GO:0032502; GO:0065007
miR-153	miR-503	GO:0009987; GO:0022610; GO:0032502; GO:0051704
miR-153	miR-505	GO:0009987; GO:0022610; GO:0032502; GO:0065007
miR-153	miR-539	GO:0008152; GO:0009987; GO:0022610; GO:0032502; GO:0065007
miR-153	miR-7	GO:0009987; GO:0022610
miR-153	miR-93.hd/291-3p/294/295/302/372/373/520	GO:0008152; GO:0009987; GO:0022610; GO:0032502; GO:0065007
miR-17-5p/20/93.mr/106/519.d	miR-181	GO:0008152; GO:0009987; GO:0022610; GO:0032501; GO:0032502; GO:0065007
miR-17-5p/20/93.mr/106/519.d	miR-185	GO:0009987; GO:0022610; GO:0032502
miR-17-5p/20/93.mr/106/519.d	miR-186	GO:0008152; GO:0009987; GO:0022610; GO:0032502; GO:0065007
miR-17-5p/20/93.mr/106/519.d	miR-19	GO:0008152; GO:0009987; GO:0022610; GO:0032502; GO:0048511; GO:0065007
miR-17-5p/20/93.mr/106/519.d	miR-218	GO:0008152; GO:0009987; GO:0022610; GO:0032502; GO:0048511; GO:0065007
miR-17-5p/20/93.mr/106/519.d	miR-221/222	GO:0008152; GO:0009987; GO:0022610; GO:0032502; GO:0065007
miR-17-5p/20/93.mr/106/519.d	miR-23	GO:0008152; GO:0009987; GO:0022610; GO:0032502; GO:0065007
miR-17-5p/20/93.mr/106/519.d	miR-320	GO:0008152; GO:0009987; GO:0022610; GO:0032502; GO:0048511; GO:0065007
miR-17-5p/20/93.mr/106/519.d	miR-329	GO:0009987; GO:0022610; GO:0032502; GO:0065007
miR-17-5p/20/93.mr/106/519.d	miR-330	GO:0008152; GO:0009987; GO:0022610; GO:0032502; GO:0048511; GO:0065007
miR-17-5p/20/93.mr/106/519.d	miR-362	GO:0009987; GO:0022610; GO:0032502
miR-17-5p/20/93.mr/106/519.d	miR-369-3p	GO:0008152; GO:0009987; GO:0022610; GO:0032502; GO:0065007
miR-17-5p/20/93.mr/106/519.d	miR-374	GO:0008152; GO:0009987; GO:0032502; GO:0065007
miR-17-5p/20/93.mr/106/	miR-495	GO:0008152; GO:0009987; GO:0022610; GO:0032502; GO:0065007

519.d		
miR-17-5p/20/93.mr/106/519.d	miR-505	GO:0009987; GO:0022610; GO:0032502
miR-17-5p/20/93.mr/106/519.d	miR-539	GO:0008152; GO:0009987; GO:0022610; GO:0032502; GO:0065007
miR-17-5p/20/93.mr/106/519.d	miR-93.hd/291-3p/294/295/302/372/373/520	GO:0008152; GO:0009987; GO:0022610; GO:0032502; GO:0065007
miR-181	miR-182	GO:0008152; GO:0009987; GO:0032502; GO:0043473; GO:0051234; GO:0051704; GO:0065007
miR-181	miR-185	GO:0009987; GO:0022610; GO:0032502
miR-181	miR-186	GO:0008152; GO:0009987; GO:0022610; GO:0032502; GO:0051704; GO:0065007
miR-181	miR-19	GO:0008152; GO:0009987; GO:0022610; GO:0032502; GO:0065007
miR-181	miR-200bc/429	GO:0008152; GO:0009987; GO:0032502; GO:0065007
miR-181	miR-218	GO:0008152; GO:0009987; GO:0022610; GO:0032502; GO:0065007
miR-181	miR-221/222	GO:0009987; GO:0022610; GO:0032502; GO:0065007
miR-181	miR-23	GO:0008152; GO:0009987; GO:0022610; GO:0032501; GO:0032502; GO:0065007
miR-181	miR-29	GO:0008152; GO:0009987; GO:0022610; GO:0032502; GO:0065007
miR-181	miR-320	GO:0008152; GO:0009987; GO:0022610; GO:0032502; GO:0065007
miR-181	miR-329	GO:0009987; GO:0022610; GO:0032502; GO:0065007
miR-181	miR-330	GO:0009987; GO:0022610; GO:0032502; GO:0065007
miR-181	miR-362	GO:0009987; GO:0022610; GO:0032502
miR-181	miR-369-3p	GO:0008152; GO:0009987; GO:0022610; GO:0032502; GO:0048511; GO:0065007
miR-181	miR-448	GO:0009987; GO:0022610; GO:0032501; GO:0032502; GO:0065007
miR-181	miR-495	GO:0008152; GO:0009987; GO:0022610; GO:0032502; GO:0065007
miR-181	miR-503	GO:0009987; GO:0022610; GO:0032502; GO:0065007
miR-181	miR-505	GO:0009987; GO:0022610; GO:0032502; GO:0051704; GO:0065007
miR-181	miR-539	GO:0008152; GO:0009987; GO:0022610; GO:0032502; GO:0065007
miR-181	miR-9	GO:0008152; GO:0009987; GO:0032502; GO:0043473; GO:0051179; GO:0065007
miR-181	miR-93.hd/291-3p/294/295/302/372/373/520	GO:0008152; GO:0009987; GO:0022610; GO:0032502; GO:0065007
miR-182	miR-200bc/429	GO:0008152; GO:0009987; GO:0032502; GO:0051179; GO:0051234; GO:0065007
miR-182	miR-27	GO:0009987; GO:0032502; GO:0043473; GO:0051234; GO:0065007
miR-182	miR-96	GO:0008152; GO:0009987; GO:0032501; GO:0032502; GO:0043473; GO:0051179; GO:0051234; GO:0065007
miR-185	miR-186	GO:0009987; GO:0022610; GO:0032502
miR-185	miR-19	GO:0009987; GO:0022610; GO:0032502; GO:0040011; GO:0065007
miR-185	miR-218	GO:0009987; GO:0022610; GO:0032502
miR-185	miR-221/222	GO:0009987; GO:0022610; GO:0032502
miR-185	miR-23	GO:0009987; GO:0022610; GO:0032502

miR-185	miR-29	GO:0009987; GO:0022610; GO:0032502
miR-185	miR-320	GO:0009987; GO:0022610; GO:0032502
miR-185	miR-329	GO:0009987; GO:0022610; GO:0032502
miR-185	miR-330	GO:0009987; GO:0022610; GO:0032502
miR-185	miR-362	GO:0009987; GO:0022610; GO:0032502
miR-185	miR-369-3p	GO:0009987; GO:0022610; GO:0032502
miR-185	miR-448	GO:0009987; GO:0022610; GO:0032502; GO:0065007
miR-185	miR-495	GO:0009987; GO:0022610; GO:0032502
miR-185	miR-503	GO:0009987; GO:0022610; GO:0032502
miR-185	miR-505	GO:0009987; GO:0022610; GO:0032502
miR-185	miR-539	GO:0009987; GO:0022610; GO:0032502
miR-185	miR-93.hd/291-3p/294/295/302/372/373/520	GO:0009987; GO:0022610; GO:0032502
miR-186	miR-19	GO:0009987; GO:0022610; GO:0032502; GO:0065007
miR-186	miR-218	GO:0009987; GO:0022610; GO:0032502; GO:0065007
miR-186	miR-221/222	GO:0009987; GO:0022610; GO:0032502
miR-186	miR-23	GO:0008152; GO:0009987; GO:0022610; GO:0032502; GO:0051704; GO:0065007
miR-186	miR-29	GO:0009987; GO:0022610; GO:0032502; GO:0051704; GO:0065007
miR-186	miR-320	GO:0008152; GO:0009987; GO:0022610; GO:0032502; GO:0051704; GO:0065007
miR-186	miR-329	GO:0009987; GO:0022610; GO:0032502
miR-186	miR-330	GO:0009987; GO:0022610; GO:0032502; GO:0065007
miR-186	miR-362	GO:0009987; GO:0022610; GO:0032502
miR-186	miR-369-3p	GO:0009987; GO:0022610; GO:0032502; GO:0065007
miR-186	miR-448	GO:0008152; GO:0009987; GO:0022610; GO:0032502; GO:0048511; GO:0065007
miR-186	miR-495	GO:0008152; GO:0009987; GO:0022610; GO:0032502; GO:0065007
miR-186	miR-503	GO:0009987; GO:0022610; GO:0032502
miR-186	miR-505	GO:0009987; GO:0022610; GO:0032502; GO:0051704; GO:0065007
miR-186	miR-539	GO:0008152; GO:0009987; GO:0022610; GO:0032502; GO:0065007
miR-186	miR-93.hd/291-3p/294/295/302/372/373/520	GO:0008152; GO:0009987; GO:0022610; GO:0032502; GO:0065007
miR-19	miR-218	GO:0009987; GO:0022610; GO:0032502; GO:0048511; GO:0065007
miR-19	miR-221/222	GO:0009987; GO:0022610; GO:0032502; GO:0065007
miR-19	miR-23	GO:0008152; GO:0009987; GO:0022610; GO:0032502; GO:0065007
miR-19	miR-29	GO:0008152; GO:0009987; GO:0022610; GO:0032502; GO:0065007
miR-19	miR-320	GO:0008152; GO:0009987; GO:0022610; GO:0032501; GO:0032502; GO:0048511; GO:0065007
miR-19	miR-329	GO:0009987; GO:0022610; GO:0032502; GO:0065007
miR-19	miR-330	GO:0008152; GO:0009987; GO:0022610; GO:0032501; GO:0032502; GO:0065007
miR-19	miR-362	GO:0009987; GO:0022610; GO:0032502
miR-19	miR-369-3p	GO:0009987; GO:0022610; GO:0032502; GO:0065007
miR-19	miR-448	GO:0009987; GO:0022610; GO:0032502; GO:0051234; GO:0065007
miR-19	miR-495	GO:0008152; GO:0009987; GO:0022610; GO:0032501;

		GO:0032502; GO:0065007
miR-19	miR-503	GO:0009987; GO:0022610; GO:0032502
miR-19	miR-505	GO:0009987; GO:0022610; GO:0032502; GO:0051234; GO:0065007
miR-19	miR-539	GO:0009987; GO:0022610; GO:0032502; GO:0065007
miR-19	miR-93.hd/291-3p/294/295/302/372/373/520	GO:0008152; GO:0009987; GO:0022610; GO:0032502; GO:0065007
miR-218	miR-221/222	GO:0009987; GO:0022610; GO:0032502; GO:0065007
miR-218	miR-23	GO:0009987; GO:0022610; GO:0032502; GO:0065007
miR-218	miR-29	GO:0008152; GO:0009987; GO:0022610; GO:0032502; GO:0051704; GO:0065007
miR-218	miR-320	GO:0008152; GO:0009987; GO:0022610; GO:0032502; GO:0065007
miR-218	miR-329	GO:0009987; GO:0022610; GO:0032502
miR-218	miR-330	GO:0009987; GO:0022610; GO:0032502; GO:0065007
miR-218	miR-362	GO:0009987; GO:0022610; GO:0032502
miR-218	miR-369-3p	GO:0009987; GO:0022610; GO:0032501; GO:0032502; GO:0065007
miR-218	miR-448	GO:0009987; GO:0022610; GO:0032502; GO:0051234; GO:0065007
miR-218	miR-495	GO:0009987; GO:0022610; GO:0032502; GO:0065007
miR-218	miR-503	GO:0009987; GO:0022610; GO:0032502; GO:0065007
miR-218	miR-505	GO:0009987; GO:0022610; GO:0032502; GO:0065007
miR-218	miR-539	GO:0009987; GO:0022610; GO:0032502; GO:0065007
miR-218	miR-93.hd/291-3p/294/295/302/372/373/520	GO:0008152; GO:0009987; GO:0022610; GO:0032502; GO:0048511; GO:0065007
miR-221/222	miR-23	GO:0009987; GO:0022610; GO:0032502; GO:0065007
miR-221/222	miR-29	GO:0009987; GO:0022610; GO:0032502
miR-221/222	miR-320	GO:0009987; GO:0022610; GO:0032502; GO:0065007
miR-221/222	miR-329	GO:0009987; GO:0022610; GO:0032502
miR-221/222	miR-330	GO:0009987; GO:0022610; GO:0032502
miR-221/222	miR-362	GO:0009987; GO:0022610; GO:0032502
miR-221/222	miR-369-3p	GO:0009987; GO:0022610; GO:0032502; GO:0065007
miR-221/222	miR-448	GO:0009987; GO:0022610; GO:0032502
miR-221/222	miR-495	GO:0008152; GO:0009987; GO:0022610; GO:0032502; GO:0065007
miR-221/222	miR-503	GO:0009987; GO:0022610; GO:0032502
miR-221/222	miR-505	GO:0009987; GO:0022610; GO:0032502
miR-221/222	miR-539	GO:0009987; GO:0022610; GO:0032502; GO:0065007
miR-221/222	miR-93.hd/291-3p/294/295/302/372/373/520	GO:0009987; GO:0022610; GO:0032502; GO:0065007
miR-23	miR-29	GO:0008152; GO:0009987; GO:0022610; GO:0032502; GO:0065007
miR-23	miR-320	GO:0008152; GO:0009987; GO:0022610; GO:0032502; GO:0065007
miR-23	miR-329	GO:0001906; GO:0009987; GO:0022610; GO:0032502; GO:0065007
miR-23	miR-330	GO:0009987; GO:0022610; GO:0032502; GO:0065007
miR-23	miR-362	GO:0009987; GO:0022610; GO:0032502
miR-23	miR-369-3p	GO:0009987; GO:0022610; GO:0032501; GO:0032502; GO:0065007
miR-23	miR-448	GO:0009987; GO:0022610; GO:0032502; GO:0065007
miR-23	miR-495	GO:0008152; GO:0009987; GO:0022610; GO:0032502;

		GO:0065007
miR-23	miR-503	GO:0009987; GO:0022610; GO:0032502
miR-23	miR-505	GO:0008152; GO:0009987; GO:0022610; GO:0032502; GO:0051704; GO:0065007
miR-23	miR-539	GO:0008152; GO:0009987; GO:0022610; GO:0032502; GO:0065007
miR-23	miR-93.hd/291-3p/294/295/302/372/373/520	GO:0001906; GO:0008152; GO:0009987; GO:0022414; GO:0022610; GO:0032502; GO:0065007
miR-26	miR-29	GO:0008152; GO:0009987; GO:0022610; GO:0032502; GO:0065007
miR-27	miR-374	GO:0008152; GO:0009987; GO:0022414; GO:0032501; GO:0032502; GO:0050896; GO:0051704; GO:0065007
miR-29	miR-320	GO:0008152; GO:0009987; GO:0022610; GO:0032502; GO:0065007
miR-29	miR-329	GO:0009987; GO:0022610; GO:0032502
miR-29	miR-330	GO:0009987; GO:0022610; GO:0032502; GO:0065007
miR-29	miR-362	GO:0009987; GO:0022610; GO:0032502
miR-29	miR-369-3p	GO:0008152; GO:0009987; GO:0022610; GO:0032502; GO:0065007
miR-29	miR-448	GO:0009987; GO:0022610; GO:0032502; GO:0065007
miR-29	miR-495	GO:0008152; GO:0009987; GO:0022610; GO:0032502; GO:0065007
miR-29	miR-503	GO:0009987; GO:0022610; GO:0032502
miR-29	miR-505	GO:0009987; GO:0022610; GO:0032502
miR-29	miR-539	GO:0008152; GO:0009987; GO:0022610; GO:0032502; GO:0065007
miR-29	miR-93.hd/291-3p/294/295/302/372/373/520	GO:0008152; GO:0009987; GO:0022610; GO:0032502; GO:0065007
miR-30-5p	miR-369-3p	GO:0008152; GO:0009987; GO:0032501; GO:0032502; GO:0048511; GO:0065007
miR-30-5p	miR-409-3p	GO:0001906; GO:0032502; GO:0043473; GO:0051234; GO:0065007
miR-30-5p	miR-448	GO:0008152; GO:0009987; GO:0032502; GO:0043473; GO:0048511; GO:0065007
miR-30-5p	miR-496	GO:0008152; GO:0009987; GO:0022610; GO:0032501; GO:0032502; GO:0043473; GO:0048511; GO:0051234; GO:0065007
miR-320	miR-329	GO:0008152; GO:0009987; GO:0022610; GO:0032502; GO:0065007
miR-320	miR-330	GO:0008152; GO:0009987; GO:0022610; GO:0032502; GO:0048511; GO:0065007
miR-320	miR-362	GO:0009987; GO:0022610; GO:0032502
miR-320	miR-369-3p	GO:0008152; GO:0009987; GO:0022610; GO:0032502; GO:0065007
miR-320	miR-448	GO:0009987; GO:0022610; GO:0032502; GO:0065007
miR-320	miR-495	GO:0008152; GO:0009987; GO:0022610; GO:0032502; GO:0048511; GO:0065007
miR-320	miR-503	GO:0009987; GO:0022610; GO:0032502; GO:0051704
miR-320	miR-505	GO:0009987; GO:0022610; GO:0032502; GO:0065007
miR-320	miR-539	GO:0008152; GO:0009987; GO:0022610; GO:0032502; GO:0065007
miR-320	miR-93.hd/291-3p/294/295/302/372/373/520	GO:0008152; GO:0009987; GO:0022610; GO:0032502; GO:0065007
miR-329	miR-330	GO:0009987; GO:0022610; GO:0032502; GO:0065007

miR-329	miR-362	GO:0009987; GO:0022610; GO:0032502
miR-329	miR-369-3p	GO:0009987; GO:0022610; GO:0032502; GO:0065007
miR-329	miR-448	GO:0009987; GO:0022610; GO:0032502
miR-329	miR-495	GO:0001906; GO:0009987; GO:0022610; GO:0032502; GO:0065007
miR-329	miR-503	GO:0009987; GO:0022610; GO:0032502; GO:0051704
miR-329	miR-505	GO:0009987; GO:0022610; GO:0032502; GO:0051704
miR-329	miR-539	GO:0009987; GO:0022610; GO:0032502
miR-329	miR-93.hd/291-3p/294/295/302/372/373/520	GO:0001906; GO:0009987; GO:0022610; GO:0032502; GO:0065007
miR-330	miR-362	GO:0009987; GO:0022610; GO:0032502
miR-330	miR-369-3p	GO:0009987; GO:0022610; GO:0032502; GO:0065007
miR-330	miR-448	GO:0001906; GO:0009987; GO:0022610; GO:0032502
miR-330	miR-495	GO:0008152; GO:0009987; GO:0022610; GO:0032502; GO:0065007
miR-330	miR-503	GO:0009987; GO:0022610; GO:0032502; GO:0051704
miR-330	miR-505	GO:0009987; GO:0022610; GO:0032502; GO:0065007
miR-330	miR-539	GO:0009987; GO:0022610; GO:0032502; GO:0065007
miR-330	miR-93.hd/291-3p/294/295/302/372/373/520	GO:0008152; GO:0009987; GO:0022610; GO:0032502; GO:0065007
miR-335	miR-7	GO:0032502; GO:0051704; GO:0065007
miR-34/449	miR-448	GO:0001906; GO:0009987; GO:0032502; GO:0043473; GO:0048511; GO:0065007
miR-362	miR-369-3p	GO:0009987; GO:0022610; GO:0032502
miR-362	miR-448	GO:0009987; GO:0022610; GO:0032502
miR-362	miR-495	GO:0009987; GO:0022610; GO:0032502
miR-362	miR-503	GO:0009987; GO:0022610; GO:0032502
miR-362	miR-505	GO:0009987; GO:0022610; GO:0032502
miR-362	miR-539	GO:0009987; GO:0022610; GO:0032502
miR-362	miR-93.hd/291-3p/294/295/302/372/373/520	GO:0009987; GO:0022610; GO:0032502
miR-369-3p	miR-374	GO:0008152; GO:0009987; GO:0032501; GO:0032502; GO:0065007
miR-369-3p	miR-448	GO:0009987; GO:0022610; GO:0032502; GO:0065007
miR-369-3p	miR-495	GO:0008152; GO:0009987; GO:0022610; GO:0032502; GO:0065007
miR-369-3p	miR-503	GO:0009987; GO:0022610; GO:0032502; GO:0065007
miR-369-3p	miR-505	GO:0009987; GO:0022610; GO:0032502; GO:0065007
miR-369-3p	miR-539	GO:0009987; GO:0022610; GO:0032502; GO:0065007
miR-369-3p	miR-543	GO:0008152; GO:0009987; GO:0032501; GO:0032502; GO:0040007; GO:0065007
miR-369-3p	miR-9	GO:0008152; GO:0009987; GO:0032502; GO:0048511; GO:0065007
miR-369-3p	miR-93.hd/291-3p/294/295/302/372/373/520	GO:0008152; GO:0009987; GO:0022610; GO:0032502; GO:0065007
miR-374	miR-410	GO:0008152; GO:0009987; GO:0032501; GO:0032502; GO:0065007
miR-374	miR-543	GO:0008152; GO:0009987; GO:0032502; GO:0040007; GO:0065007
miR-380-5p	miR-496	GO:0032501; GO:0032502; GO:0043473; GO:0048511; GO:0065007
miR-381	miR-493-5p	GO:0008152; GO:0009987; GO:0032502; GO:0051704;

		GO:0065007
miR-381	miR-496	GO:0008152; GO:0009987; GO:0032501; GO:0032502; GO:0043473; GO:0065007
miR-448	miR-495	GO:0008152; GO:0009987; GO:0022610; GO:0032502; GO:0065007
miR-448	miR-503	GO:0009987; GO:0022414; GO:0022610; GO:0032502; GO:0051704
miR-448	miR-505	GO:0009987; GO:0022610; GO:0032502; GO:0065007
miR-448	miR-539	GO:0009987; GO:0022610; GO:0032502; GO:0065007
miR-448	miR-7	GO:0009987; GO:0022610
miR-448	miR-93.hd/291-3p/294/295/302/372/373/520	GO:0008152; GO:0009987; GO:0022610; GO:0032502; GO:0065007
miR-495	miR-503	GO:0009987; GO:0022610; GO:0032502; GO:0065007
miR-495	miR-505	GO:0009987; GO:0022610; GO:0032502; GO:0065007
miR-495	miR-539	GO:0008152; GO:0009987; GO:0022610; GO:0032502; GO:0065007
miR-495	miR-93.hd/291-3p/294/295/302/372/373/520	GO:0008152; GO:0009987; GO:0022610; GO:0032502; GO:0065007
miR-503	miR-505	GO:0009987; GO:0022610; GO:0032502
miR-503	miR-539	GO:0009987; GO:0022610; GO:0032502
miR-503	miR-93.hd/291-3p/294/295/302/372/373/520	GO:0009987; GO:0022610; GO:0032502; GO:0065007
miR-505	miR-539	GO:0009987; GO:0022610; GO:0032502
miR-505	miR-93.hd/291-3p/294/295/302/372/373/520	GO:0009987; GO:0022610; GO:0032502
miR-539	miR-93.hd/291-3p/294/295/302/372/373/520	GO:0008152; GO:0009987; GO:0022610; GO:0032502; GO:0065007
