# Supplementary material for: Coregulation of transcription factors and microRNAs in human transcriptional regulatory network
Source: BMC Bioinformatics. 2011 Feb 15;12(Suppl 1):S41. doi: 10.1186/1471-2105-12-S1-S41 (PMC3044298; doi:10.1186/1471-2105-12-S1-S41)
Supplement: Additional file 2 — List of network motifs for function-enriched coregulation pairshttp://idv.sinica.edu.tw/joeychen/APBC2011/AdditionalFile2.pdf [file 1471-2105-12-S1-S41-S2.pdf]

A. Enriched Motifs in TF-TF co-regulation

A.1 Bidirectional Feed-Forward Loop

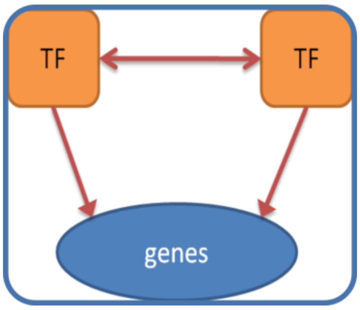

Regulator A (Left TF)	Regulator B (Right TF)	Validated Interaction*	Number of Shared Targets
AR	NFKB1	←	39
AR	RARA	←→	102
AR	RELA	←→	20
CEBPA	TP53	←→	30
E2F4	TP53		31
EGR1	JUN	←→	24
EGR1	TP53	←→	23
EGR1	WT1	←→	12
ETS1	ETS2	←→	37
ETS1	NFKB1	←	49
ETS1	TP53	←→	36
ETS2	TP53	←→	16
MYC	TP53	←	41
NFIC	TP53	←→	15
NFKB1	REL		22
NFKB1	TP53	→	40
NFKB2	RELA	→	6
RELA	TP53	←→	26

*Experimental evidences existed for the given direction(s) of the regulation between the TF pairs.

A.2 Unidirectional Feed-Forward Loop

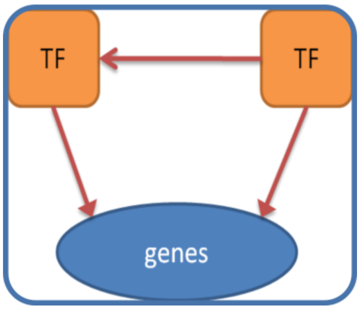

Regulator A (Left TF)	Regulator B (Right TF)	Validated Interaction	Number of Shared Targets
AR	EGR1	←	32
AR	SP1	←	51
ATF1	CEBPA		10
CEBPA	MYB	←	27
CEBPA	USF1	←	17
CEBPA	USF2	←	11
CEBPB	CREB1	←	13
CEBPB	EGR1	←	10
CREB1	CEBPA	←	27
CREB1	MYC		29
EGR1	ATF1	←	8
EGR1	CREB1	←	20
EGR1	ETS1	←	18
EGR1	SP1	←	45
ELK1	SP1	←	5
ESR1	TP53	←	21
ETS1	AR	←	17
ETS1	JUN	←	53
ETS1	POU2F1	←	25
ETS1	RARA	←	9
ETS1	SP1	←	53
ETS2	POU2F1	←	10
ETS2	SP1	←	24
FOS	CEBPB	←	8
FOS	JUN	←	34
FOS	NFKB1	←	18

JUN	CREB1	←	40
JUN	REL	←	9
JUN	SP1	←	70
MYB	ETS1		25
MYB	PPARG		6
MYC	ETS1	←	25
MYC	NFKB1	←	30
MYC	PPARG	←	14
MYC	RELA	←	12
MYC	SP1	←	54
NFKB1	CEBPA		40
NFKB1	CEBPB		26
NFKB1	EGR1		32
NFKB1	RARA		18
NFKB1	RARB		10
NFKB1	RARG		7
NFKB1	RELA		65
NFKB1	SPI1		19
POU2F1	AR	←	15
RARA	SP1	←	29
RARB	PPARG	←	8
REL	SP1		8
REL	SPI1		5
RELA	EGR1	←	16
RELA	SP1	←	28
SMAD3	SP1		18
SP1	CEBPA		53
SP1	MYB		36
SP1	POU2F1		26
SP1	TFAP2A		83
SPI1	JUN	←	15
SPI1	POU2F1	←	9
SPI1	SP1	←	28
STAT3	AR	←	14
STAT3	ATF1	←	9
STAT3	CREB1	←	16
STAT3	TP53	←	14

TFAP2A	POU2F1	←	19
TP53	JUN	←	31
WT1	NFKB1	←	8
WT1	RELA	←	5
WT1	SP1	←	19

A.3 Common Upstream TF

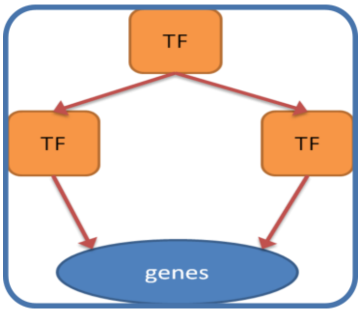

Upstream Regulator (Top TF)	Regulator A (Left TF)	Regulator B (Right TF)	Number of Shared Targets
EGR1	AR	CEBPB	17
SP1	AR	EGR1	32
AR/BRCA1/NFKB1/RARA/SMAD4/SP1	AR	ETS1	17
AR/EGR1/NFKB1/RARA/RELA	AR	NFKB1	39
AR	AR	POU2F1	15
AR/RARA/SP1	AR	RARA	102
NFKB1/SP1	AR	REL	9
AR/EGR1/SP1	AR	RELA	20
SP1	AR	SPI1	9
AR/BRCA1/RARA	AR	STAT3	14
BRCA1/EGR1/NFKB1/RELA	AR	TP53	34
CEBPA	ATF1	CREB1	52
CEBPA	ATF1	E2F1	7
CEBPA	ATF1	NFKB1	16
CEBPA	ATF1	SP1	24
CEBPA	ATF1	TP53	18
ATF1/CREB1/ETS2/SP1/TP53	BRCA1	EGR1	8
SP1/TP53	BRCA1	RELA	5
MYC	CEBPA	CREB1	27
MYB/TP53	CEBPA	EGR1	24

TP53	CEBPA	ETS1	27
USF1	CEBPA	ETV4	13
TP53	CEBPA	FOS	16
MYC	CEBPA	JUN	35
MYB	CEBPA	MYB	27
TP53	CEBPA	NFIC	20
TP53	CEBPA	NFKB1	40
MYC	CEBPA	RARA	16
TP53	CEBPA	RELA	23
MYB/USF1/USF2	CEBPA	SP1	53
TP53	CEBPA	STAT3	14
MYC/TP53/USF1	CEBPA	TP53	30
MYB/MYC	CEBPA	WT1	6
CREB1	CEBPB	EGR1	10
STAT3	CEBPB	FOS	8
CREB1/EGR1/STAT3	CEBPB	JUN	20
STAT3	CEBPB	MYC	6
EGR1	CEBPB	NFKB1	26
EGR1	CEBPB	RELA	17
EGR1	CEBPB	TP53	16
CEBPA/MYC	CREB1	E2F1	9
MYC	CREB1	JUN	40
MYC	CREB1	MYC	29
CEBPA	CREB1	NFKB1	35
MYC	CREB1	RARA	20
CEBPA	CREB1	SP1	50
CEBPA/MYC	CREB1	TP53	21
CEBPA/TP53	E2F1	NFKB1	17
TP53	E2F4	TP53	31
ETS1/ETS2/JUN/PAX5/SP1/TP53	EGR1	ETS1	18
ATF1/CREB1/ETS2/JUN/SP1	EGR1	JUN	24
ETS1/ETS2/JUN/MYB/SP1/TFAP2A/TP53	EGR1	MYC	22
ETS1/TP53	EGR1	NFKB1	32
SP1/WT1	EGR1	RARA	19
SP1/TP53	EGR1	RELA	16
MYB/TFAP2A	EGR1	SP1	45
ATF1/CREB1/TP53	EGR1	STAT3	12

ETS1/ETS2/JUN/PAX5/TP53/WT1	EGR1	TP53	23
MYB/SP1/WT1	EGR1	WT1	12
CEBPA/MYB	ELK1	SP1	5
BRCA1/CEBPA/EGR1/MYC/NFKB1/PAX5	ELK1	TP53	6
AR/SP1/TP53	ESR1	RELA	9
BRCA1/HIF1A/JUN/PAX5/TP53/USF1	ESR1	TP53	21
ETS1/ETV4/JUN/POU2F1/SP1/TP53	ETS1	ETS2	37
AR/ETS2/JUN/RARA/SMAD4/SP1	ETS1	JUN	53
AR/ETS1/ETS2/ETV4/JUN/NFKB1	ETS1	MYB	25
AR/ETS1/ETS2/JUN/NFKB1/POU2F1/POU2F2/SP1/TP53	ETS1	MYC	25
AR/ETS1/NFKB1/RARA/TP53	ETS1	NFKB1	49
AR	ETS1	POU2F1	25
AR/RARA	ETS1	PPARG	10
AR/RARA/SP1	ETS1	RARA	9
AR/RARA	ETS1	RARB	8
AR/SP1/TP53	ETS1	RELA	21
POU2F1	ETS1	SP1	53
JUN/POU2F1/POU2F2/SP1	ETS1	SPI1	28
BRCA1/ETS1/ETS2/FOS/JUN/NFKB1/PAX2/PAX5/TP53	ETS1	TP53	36
ETV4/SP1	ETS1	USF1	14
POU2F1	ETS2	SP1	24
ETS1/JUN/MYC/TP53	ETS2	TP53	16
ETS1	ETV4	NFKB1	23
ETS1	ETV4	TFAP2A	27
ETS1/USF1	ETV4	TP53	13
ATF1/ATF2/BCL6/CREM/ESR1/ESR2/ETS2/JUN/NFIC/STAT1/STAT3	FOS	JUN	34
BCL6/CEBPB/ETS1/NFKB1/TP53	FOS	NFKB1	18
NFKB1	FOS	REL	5
TP53	FOS	RELA	10
ESR1	FOS	SP1	17
SP1	JUN	JUND	12
SMAD3/SP1	JUN	LEF1	8
AR/BCL6/E2F4/EGR1/RARA/RARB/RARG/REL	JUN	NFKB1	71

E2F4/SP1	JUN	REL	9
AR/EGR1/SP1	JUN	RELA	28
E2F4/ESR1	JUN	SP1	70
JUN/SP1	JUN	SPI1	15
BCL6/E2F4/EGR1/ETS2/JUN/MYC/NFIC/STAT1	JUN	TP53	31
AR/EGR1/ESR1	MYB	PPARG	6
AR/PPARG	MYB	RARB	6
AR/EGR1/PPARD	MYB	RELA	12
ESR1/MYB/TFAP2A	MYB	SP1	36
AR	MYB	STAT3	7
EGR1/ETS1/ETS2/JUN/NFKB1/WT1	MYB	TP53	31
AR/E2F4/ETS1/NFKB1/RARB/RELA/TP53	MYC	NFKB1	30
AR/ESR1	MYC	PPARG	14
AR/PPARD/SP1/TP53	MYC	RELA	12
E2F4/ESR1/MYB/MYBL2/POU2F1/TFAP2A	MYC	SP1	54
E2F4/ETS1/ETS2/JUN/MYC/NFIC/NFKB1/RELA/STAT1/TP53	MYC	TP53	41
TP53	NFIC	RELA	14
POU2F1	NFIC	SP1	31
TP53	NFIC	TP53	15
BCL3/E2F4/RELA	NFKB1	NFKB2	5
AR	NFKB1	POU2F1	25
AR/CEBPA/CEBPB/EGR1/RARA	NFKB1	PPARG	18
AR/RARA	NFKB1	RARA	18
AR/RARA/RARB/RARG	NFKB1	RARB	10
AR/RARA	NFKB1	RARG	7
E2F4/NFKB1/SPI1	NFKB1	REL	22
AR/EGR1/TP53	NFKB1	RELA	65
CEBPA/E2F4	NFKB1	SP1	65
SPI1	NFKB1	SPI1	19
TP53	NFKB1	STAT1	23
AR/RARA/TP53	NFKB1	STAT3	23
CEBPA/ETS1	NFKB1	TFAP2A	41
BCL6/CEBPA/E2F4/EGR1/ETS1/HIF1A/NFKB1/RELA/TP53	NFKB1	TP53	40
EGR1/NFKB1/RELA	NFKB1	WT1	8

SP1	NFKB2	RELA	6
AR	POU2F1	RARA	10
AR	POU2F1	RELA	12
AR/CREB1/RARA	PPARG	RARB	8
AR/EGR1	PPARG	RELA	9
CEBPA/ESR1	PPARG	SP1	21
AR/CREB1/RARA	PPARG	STAT3	9
ESR1/ESR2	PPARG	STAT5A	8
CEBPA/EGR1	PPARG	TP53	14
SP1	RARA	REL	6
AR/SP1	RARA	RELA	10
ESR1	RARA	SP1	29
MYC/WT1	RARA	TP53	21
AR	RARB	RELA	10
MYC	RARB	TP53	15
AR	RARG	RELA	6
SP1	REL	RELA	22
E2F4/POU2F1	REL	SP1	8
POU2F1/POU2F2/SP1/SPI1	REL	SPI1	5
E2F4/NFKB1	REL	TP53	8
SP1	RELA	SMAD4	6
SP1	RELA	SPI1	7
TP53	RELA	STAT1	10
AR/TP53	RELA	STAT3	12
EGR1/TP53	RELA	TP53	26
EGR1/SP1	RELA	WT1	5
POU2F1	SP1	SPI1	28
CEBPA/POU2F1	SP1	TFAP2A	83
CEBPA/E2F4/USF1	SP1	TP53	41
MYB	SP1	WT1	19
BRCA1/TP53	STAT3	TP53	14
CEBPA/ETS1/NFIC	TFAP2A	TP53	28
SP1	USF1	USF2	78

A.4 Common Upstream TF & miRNA

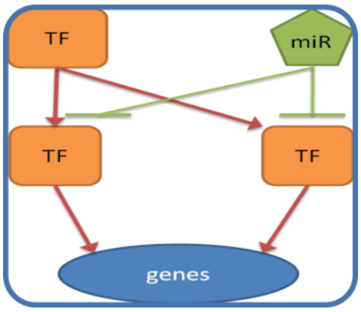

Upstream TF (Top TF)	Upstream miRNA (Top miRNA)	Regulator A (Left TF)	Regulator B (Right TF)	Number of Shared Targets
CEBPA	miR-9	ATF1	NFKB1	16
CEBPA	miR-410	ATF1	SP1	24
MYC	miR-124/506/miR-182/miR-25/32/92/363/367/miR-96	CEBPA	CREB1	27
MYB/TP53	miR-124/506	CEBPA	EGR1	24
TP53	miR-101/miR-124/506/miR-144/miR-326/miR-381	CEBPA	ETS1	27
TP53	miR-101/miR-144	CEBPA	FOS	16
MYC	miR-25/32/92/363/367	CEBPA	JUN	35
MYB	miR-101/miR-144/miR-182/miR-96	CEBPA	MYB	27
MYB/USF1/USF2	miR-124/506/miR-25/32/92/363/367/miR-326/miR-381	CEBPA	SP1	53
TP53	miR-124/506	CEBPA	STAT3	14
CREB1	miR-191	CEBPB	EGR1	10
MYC	miR-25/32/92/363/367	CREB1	JUN	40
MYC	miR-34b	CREB1	MYC	29
CEBPA	miR-323	CREB1	NFKB1	35
MYC	miR-128/miR-27	CREB1	RARA	20
CEBPA	miR-124.1/miR-124/506/miR-128/miR-25/32/92/363/367/miR-27	CREB1	SP1	50
ETS1/ETS2/JUN/PAX5/SP1/TP53	miR-124.1/miR-124/506/miR-181/miR-377	EGR1	ETS1	18

ATF1/CREB1/ETS2/JUN/SP1	miR-30-3p	EGR1	JUN	24
ETS1/TP53	miR-30-3p	EGR1	NFKB1	32
MYB/TFAP2A	miR-124.1/miR-124/506	EGR1	SP1	45
ATF1/CREB1/TP53	miR-124.1/miR-124/506	EGR1	STAT3	12
CEBPA/MYB	miR-135/miR-326	ELK1	SP1	5
ETS1/ETV4/JUN/POU2F1/SP1/TP53	miR-145/miR-199/miR-221/222	ETS1	ETS2	37
AR/ETS2/JUN/RARA/SMAD4/SP1	miR-139/miR-495	ETS1	JUN	53
AR/ETS1/ETS2/ETV4/JUN/NFKB1	miR-101/miR-144/miR-155/miR-200bc/429/miR-34b/miR-499	ETS1	MYB	25
AR/ETS1/ETS2/JUN/NFKB1/POU2F1/POU2F2/SP1/TP53	miR-34b	ETS1	MYC	25
AR/ETS1/NFKB1/RARA/TP53	miR-9	ETS1	NFKB1	49
AR/RARA	miR-1/206/miR-101/miR-144	ETS1	RARB	8
POU2F1	miR-124.1/miR-124/506/miR-125/351/miR-193/miR-326/miR-33/miR-378/miR-381/miR-410/miR-495	ETS1	SP1	53
JUN/POU2F1/POU2F2/SP1	miR-155	ETS1	SPI1	28
POU2F1	miR-22	ETS2	SP1	24
ATF1/ATF2/BCL6/CREM/ESR1/ESR2/ETS2/JUN/NFIC/STAT1/STAT3	miR-139	FOS	JUN	34
ESR1	miR-29	FOS	SP1	17
AR/BCL6/E2F4/EGR1/RARA/RARB/RARG/REL	miR-30-3p	JUN	NFKB1	71
E2F4/ESR1	miR-25/32/92/363/367/miR-493-5p/miR-495	JUN	SP1	70

AR/EGR1/ESR1	miR-130/301	MYB	PPARG	6
AR/PPARG	miR-101/miR-130/301/miR-144/miR-15/16/195/424/497	MYB	RARB	6
AR/RARA/RARB/RARG	miR-30-3p	NFKB1	RARB	10
AR/CREB1/RARA	miR-130/301	PPARG	RARB	8
CEBPA/ESR1	miR-128/miR-27	PPARG	SP1	21
ESR1	miR-128/miR-135/miR-218/miR-27	RARA	SP1	29
CEBPA/POU2F1	miR-135/miR-25/32/92/363/367	SP1	TFAP2A	83

A.5 Upstream Crosstalk

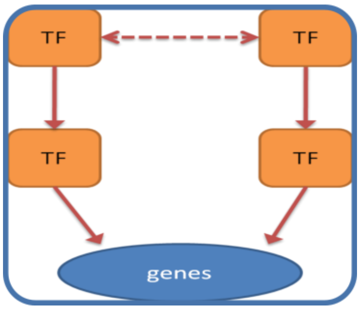

Regulator A (Left TF)	Regulator B (Right TF)	Direction of crosstalk between upstream TFs	Number of Shared Targets
AR	ATF1	←	11
AR	CEBPA	←→	30
AR	CEBPB	←→	17
AR	CREB1	←→	28
AR	EGR1	←→	32
AR	ETS1	←→	17
AR	NFKB1	←→	39
AR	POU2F1	←→	15
AR	RARA	←→	102
AR	REL	←→	9
AR	RELA	←→	20
AR	SP1	←→	51
AR	SPI1	←→	9

AR	STAT3	←→	14
AR	STAT5A	←→	10
AR	TP53	←→	34
ATF1	CEBPA	←→	10
ATF1	CEBPB	→	8
ATF1	CREB1	←	52
ATF1	E2F1	←→	7
ATF1	EGR1	←→	8
ATF1	ETS1	←→	18
ATF1	NFKB1	←→	16
ATF1	RARA	←→	7
ATF1	REL	→	5
ATF1	RELA	←→	9
ATF1	SP1	←→	24
ATF1	SPI1	→	6
ATF1	STAT3	←→	9
ATF1	TP53	←→	18
BRCA1	EGR1	←→	8
BRCA1	RELA	←→	5
CEBPA	CEBPB	←→	39
CEBPA	CREB1	←→	27
CEBPA	EGR1	←→	24
CEBPA	ETS1	←→	27
CEBPA	ETV4	←→	13
CEBPA	FOS	←→	16
CEBPA	JUN	←→	35
CEBPA	JUND	←→	11
CEBPA	MYB	←→	27
CEBPA	NFIC	←→	20
CEBPA	NFKB1	←→	40
CEBPA	POU2F1	←	16
CEBPA	RARA	←→	16
CEBPA	RARG	←→	6
CEBPA	REL	←→	9
CEBPA	RELA	←→	23
CEBPA	SP1	←→	53
CEBPA	SPI1	←→	23

CEBPA	STAT3	←→	14
CEBPA	TP53	←→	30
CEBPA	USF1	←→	17
CEBPA	USF2	←→	11
CEBPA	WT1	←→	6
CEBPB	CREB1	←→	13
CEBPB	EGR1	←→	10
CEBPB	ETS1	←→	13
CEBPB	ETV4	←	7
CEBPB	FOS	←→	8
CEBPB	JUN	←→	20
CEBPB	JUND	←	7
CEBPB	MYC	←→	6
CEBPB	NFIC	←→	12
CEBPB	NFKB1	←→	26
CEBPB	POU2F1	←→	9
CEBPB	RARA	←→	13
CEBPB	REL	←→	10
CEBPB	RELA	←→	17
CEBPB	SP1	←→	21
CEBPB	SPI1	←→	11
CEBPB	TFAP2A	←	12
CEBPB	TP53	←→	16
CEBPB	USF1	←	6
CEBPB	USF2	←	5
CREB1	E2F1	←→	9
CREB1	EGR1	←→	20
CREB1	ETS1	←→	29
CREB1	JUN	←→	40
CREB1	MYC	←→	29
CREB1	NFKB1	←→	35
CREB1	RARA	←→	20
CREB1	REL	←→	7
CREB1	RELA	←→	16
CREB1	SP1	←→	50
CREB1	SPI1	←→	7
CREB1	STAT3	←→	16

CREB1	STAT5A	←	6
CREB1	TP53	←→	21
CREB1	USF1	←→	17
E2F1	NFKB1	←→	17
E2F4	TP53	←→	31
EGR1	ETS1	←→	18
EGR1	JUN	←→	24
EGR1	MYC	←→	22
EGR1	NFKB1	←→	32
EGR1	RARA	←→	19
EGR1	RELA	←→	16
EGR1	SP1	←→	45
EGR1	STAT3	←→	12
EGR1	STAT5A	←→	7
EGR1	TP53	←→	23
EGR1	WT1	←→	12
ELK1	SP1	←→	5
ELK1	TP53	←→	6
ESR1	RELA	←→	9
ESR1	TFAP2A	←→	18
ESR1	TP53	←→	21
ETS1	ETS2	←→	37
ETS1	JUN	←→	53
ETS1	MYB	←→	25
ETS1	MYC	←→	25
ETS1	NFKB1	←→	49
ETS1	POU2F1	←→	25
ETS1	PPARG	←→	10
ETS1	RARA	←→	9
ETS1	RARB	←→	8
ETS1	RELA	←→	21
ETS1	SP1	←→	53
ETS1	SPI1	←→	28
ETS1	TP53	←→	36
ETS1	USF1	←→	14
ETS2	POU2F1	←→	10
ETS2	SP1	←→	24

ETS2	TP53	←→	16
ETV4	NFKB1	←→	23
ETV4	POU2F1	←	10
ETV4	TFAP2A	←→	27
ETV4	TP53	←→	13
FOS	JUN	←→	34
FOS	NFKB1	←→	18
FOS	REL	←→	5
FOS	RELA	←→	10
FOS	SP1	←→	17
JUN	JUND	←→	12
JUN	LEF1	←→	8
JUN	NFKB1	←→	71
JUN	REL	←→	9
JUN	RELA	←→	28
JUN	SP1	←→	70
JUN	SPI1	←→	15
JUN	TP53	←→	31
JUND	NFKB1	←→	8
MYB	PPARG	←→	6
MYB	RARB	←→	6
MYB	RELA	←→	12
MYB	SP1	←→	36
MYB	STAT3	←→	7
MYB	TP53	←→	31
MYBL2	TFAP2A	←→	6
MYC	NFKB1	←→	30
MYC	PPARG	←→	14
MYC	RELA	←→	12
MYC	SP1	←→	54
MYC	TP53	←→	41
NFIC	RELA	←→	14
NFIC	SP1	←→	31
NFIC	TP53	←→	15
NFKB1	NFKB2	←→	5
NFKB1	POU2F1	←→	25
NFKB1	PPARG	←→	18

NFKB1	RARA	←→	18
NFKB1	RARB	←→	10
NFKB1	RARG	←→	7
NFKB1	REL	←→	22
NFKB1	RELA	←→	65
NFKB1	SP1	←→	65
NFKB1	SPI1	←→	19
NFKB1	STAT1	←→	23
NFKB1	STAT3	←→	23
NFKB1	STAT5A	←→	13
NFKB1	STAT6	←→	10
NFKB1	TFAP2A	←→	41
NFKB1	TP53	←→	40
NFKB1	WT1	←→	8
NFKB2	RELA	←→	6
POU2F1	RARA	←→	10
POU2F1	RELA	←→	12
POU2F1	SP1	←→	26
POU2F1	SPI1	←→	9
POU2F1	TFAP2A	←→	19
POU2F1	TP53	←→	12
POU2F1	USF1	←	11
POU2F1	USF2	←	8
PPARG	RARB	←→	8
PPARG	RELA	←→	9
PPARG	SP1	←→	21
PPARG	STAT3	←→	9
PPARG	STAT5A	←→	8
PPARG	TP53	←→	14
RARA	REL	←→	6
RARA	RELA	←→	10
RARA	SP1	←→	29
RARA	TP53	←→	21
RARB	RELA	←→	10
RARB	SP1	←→	13
RARB	TP53	←→	15
RARG	RELA	←→	6

RARG	TP53	←→	11
REL	RELA	←→	22
REL	SP1	←→	8
REL	SPI1	←→	5
REL	STAT1	←→	6
REL	STAT5A	←→	5
REL	TP53	←→	8
RELA	SMAD4	←→	6
RELA	SP1	←→	28
RELA	SPI1	←→	7
RELA	STAT1	←→	10
RELA	STAT3	←→	12
RELA	STAT5A	←→	7
RELA	TFAP2A	←→	10
RELA	TP53	←→	26
RELA	WT1	←→	5
SMAD3	SP1	←→	18
SP1	SPI1	←→	28
SP1	STAT3	←→	22
SP1	TFAP2A	←→	83
SP1	TP53	←→	41
SP1	WT1	←→	19
STAT3	STAT5A	←→	17
STAT3	TP53	←→	14
STAT5A	TP53	←→	7
TFAP2A	TP53	←→	28

B. Enriched Motifs in TF-miRNA co-regulation

B.1 Feed-Forward Loop

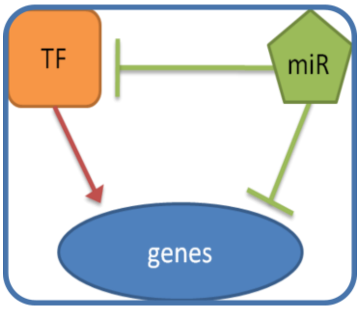

TF	miRNA	Validated Interaction*	Number of Shared Targets
CREB1	miR-181		13
CREB1	miR-182		10
CREB1	miR-27		12
CREB1	miR-96		9
ETS1	miR-181		10
SP1	miR-124/506	⊢	20
SP1	miR-149		6
SP1	miR-493-5p		9

*Annotated from DIANA TarBase v.5c

Common Upstream TF

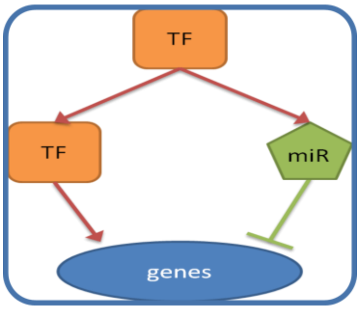

Upstream Regulator (Top TF)	Regulator A (Left TF)	Regulator B (Right miRNA)	Number of Shared Targets
NFKB1	AR	miR-15/16/195/424/497	14
CEBPA	ATF1	miR-205	5

CEBPA	CREB1	miR-205	5
CEBPA	CREB1	miR-26	9
CEBPA	CREB1	miR-27	12
PGR	MYB	miR-181	13
MYB	MYB	miR-30-5p	9
CEBPA	NFKB1	miR-30-5p	14
STAT5B	RARA	miR-493-5p	5
E2F4/MYB	SP1	miR-15/16/195/424/497	20
CEBPA/MYB	SP1	miR-30-5p	19
E2F4/NFKB1	TP53	miR-15/16/195/424/497	15
PAX8	TP53	miR-153	9
CEBPA	TP53	miR-30-5p	14
E2F4/TP53	TP53	miR-34/449	8

B.2 Upstream Crosstalk

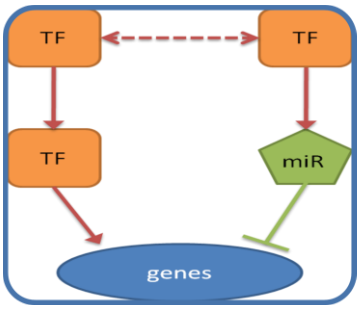

Regulator A (Left TF)	Regulator B (Right TF)	Direction of crosstalk between upstream TFs	Number of Shared Targets
AR	miR-15/16/195/424/497	←→	14
ATF1	miR-205	→	5
CREB1	miR-181	←	13
CREB1	miR-182	→	10
CREB1	miR-205	←→	5
CREB1	miR-26	→	9
CREB1	miR-27	→	12
CREB1	miR-96	→	9
EGR1	miR-493-5p	←	7
ETS1	miR-181	←→	10
ETS1	miR-30-5p	←→	12
ETS1	miR-503	←→	5

ETS2	miR-181	←→	9
MYB	miR-181	←→	13
MYB	miR-30-5p	←→	9
NFKB1	miR-143	→	5
NFKB1	miR-30-5p	→	14
NFKB1	miR-493-5p	←→	5
NFKB1	miR-503	←→	6
RARA	miR-30-5p	←→	6
RARA	miR-493-5p	←→	5
RARB	miR-493-5p	←→	5
SP1	miR-15/16/195/424/497	←→	20
SP1	miR-182	→	9
SP1	miR-30-5p	←→	19
SP1	miR-493-5p	←	9
SP1	miR-96	→	9
TP53	miR-15/16/195/424/497	←→	15
TP53	miR-153	←→	9
TP53	miR-181	←→	11
TP53	miR-204/211	→	6
TP53	miR-30-5p	←→	14
TP53	miR-34/449	←→	8
TP53	miR-96	←→	13
